# Functional and structural organization of medial entorhinal cortex layer VI

**DOI:** 10.1016/j.isci.2025.112207

**Published:** 2025-03-12

**Authors:** Märt Rannap, Shinya Ohara, Janis Winterstein, Fabian C. Roth, Andreas Draguhn, Alexei V. Egorov

**Affiliations:** 1 Institute of Physiology and Pathophysiology, Medical Faculty, Heidelberg University, 69120 Heidelberg, Germany; 2 Laboratory of Systems Neuroscience, Tohoku University Graduate School of Life Sciences, Sendai 980-8577, Japan; 3 Division of Physiology, Department of Molecular Medicine, Institute of Basic Medical Sciences, University of Oslo, 0372 Oslo, Norway; 4 PRESTO, Japan Science and Technology Agency (JST), Tokyo 102-0076, Japan

**Keywords:** Natural sciences, Biological sciences, Neuroscience, Systems neuroscience

## Abstract

Deep layers (V/VI) of the entorhinal cortex transfer hippocampal neuronal activity to downstream neocortical networks. In addition, neurons in layer VI (LVI) of the medial entorhinal cortex (MEC) project back to all hippocampal subregions and contribute to spatial coding and memory. Their role in the processing of hippocampal output signals and their interaction with LV neurons is, however, unknown. We show that spontaneously occurring hippocampal sharp wave-ripple complexes reliably propagate from area CA1 to MEC LVI. Using anterograde tracing and *in vitro* optogenetics, we confirm direct hippocampal projections to LVI and show that these follow a parallel dorsoventral topography. Further investigation of the MEC deep layer network revealed very sparse excitatory connections between LVI and LVb or LVI and LVa neurons in both directions. Together, our results establish organizational principles for the hippocampal-MEC LVI output circuit and suggest largely parallel signal processing through different cellular subpopulations in MEC deep layers.

## Highlights

• Hippocampal SPW-R reliably propagate to MEC LVI excitatory neurons
• Hippocampal projections to MEC LVI are topographically organized
• Connectivity between MEC LVI and LVa or LVb excitatory neurons is very sparse
• Signals in MEC deep layers are processed in largely parallel streams of activity

## Graphical abstract

**Figure:**
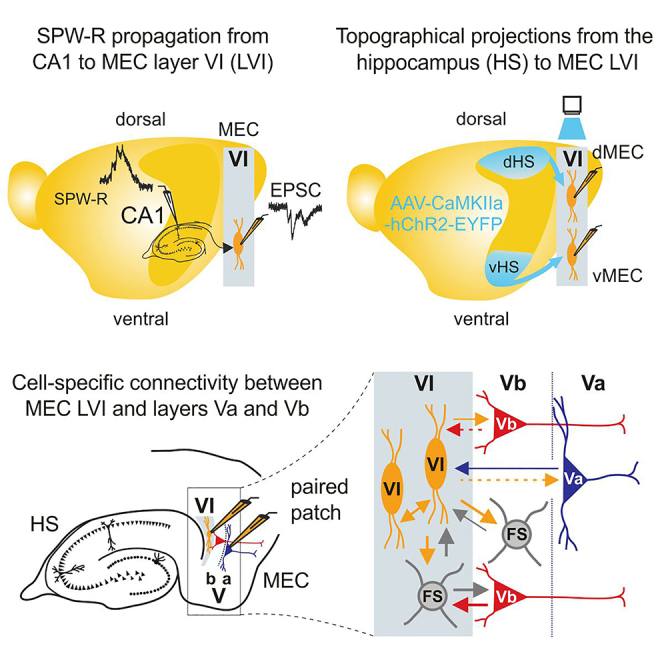

## Introduction

The entorhinal cortex (EC) and the hippocampus form a major neuronal circuit that is critically involved in spatial navigation, episodic memory, and learning.^1^ To this end the two structures integrate higher-order multimodal sensory signals, which first converge on neurons in superficial layers of EC. These provide input to all subfields of the hippocampus.^2^^,^^3^ Efferent hippocampal fibers, in turn, terminate in the deep layers V (LV) and VI (LVI) of EC, from where signals are conveyed to different downstream targets, including the neocortex.^3^^,^^4^^,^^5^^,^^6^^,^^7^ This hippocampal-entorhinal-neocortical output pathway provides a neural substrate for long-term memory consolidation.^8^^,^^9^^,^^10^

Convergent evidence from recent years shows that the deep layers of EC feature a complex structural and functional organization, which is likely to control the hippocampal-neocortical dialogue. Entorhinal LV can be divided into two sublayers: Va (LVa) and Vb (LVb). Output to the neocortex appears to originate exclusively from LVa neurons, whereas LVb neurons project locally within EC, primarily targeting superficial layers.^11^^,^^12^^,^^13^ Furthermore, hippocampal projections to LV in the medial EC (MEC) vary along the dorsoventral axis of the hippocampal formation. While the dorsal hippocampus projects preferentially to dorsal MEC LVb, ventral hippocampal neurons innervate MEC LVa neurons in both the dorsal and ventral MEC.^13^^,^^14^^,^^15^ These findings suggest considerable organizational complexity for MEC LV and indicate that MEC deep layers can support processing beyond the simple relaying of hippocampal output signals.

Only recently has the deepest layer of EC, LVI, gained increasing attention, despite its clearly documented role in hippocampal-entorhinal interactions. This layer harbors a high fraction of excitatory neurons,^2^^,^^16^ which have been shown to respond to stimulation of the hippocampal output loop.^17^^,^^18^ LVI neurons project to the thalamus,^19^^,^^20^ and, according to a recent study, to all subfields of the hippocampus.^20^ The same study revealed the importance of LVI for information processing and memory formation. While it is known that LVI receives excitatory input from hippocampal area CA1 and the subiculum,^4^^,^^6^^,^^20^^,^^21^ many important questions remain unanswered: how do hippocampal network activity patterns, such as sharp wave-ripple complexes (SPW-R), impact the receiving neurons in LVI? Are there dorsal-ventral gradients in connectivity, similar to the hippocampal output to LV? How are LVI neurons integrated into the MEC deep layer circuitry?

Here, we report that spontaneously occurring SPW-R reliably propagate from area CA1 to excitatory neurons in MEC LVI. Using anterograde tracing and *in vitro* optogenetic stimulation, we confirm direct hippocampal projections to LVI and show that these follow a similar dorsoventral topography as hippocampal projections to MEC LVb. Compared to LV, however, LVI neurons receive weaker input in both the dorsal and ventral MEC. We further investigate how LVI neurons are integrated into MEC deep layer microcircuitry, finding very sparse excitatory connections between LVI and LVb or LVI and LVa neurons. In contrast, we find strong bidirectional interlaminar connectivity between principal cells and fast-spiking inhibitory interneurons in layers VI and Vb, suggesting that the layers predominantly communicate via inhibitory interneurons. Together, our results establish organizational principles for the hippocampal-MEC LVI output circuit and indicate that MEC deep layers process hippocampal output signals in parallel streams of activity.

## Results

### SPW-R propagate efficiently to MEC layer VI excitatory neurons

**Figure 1:**
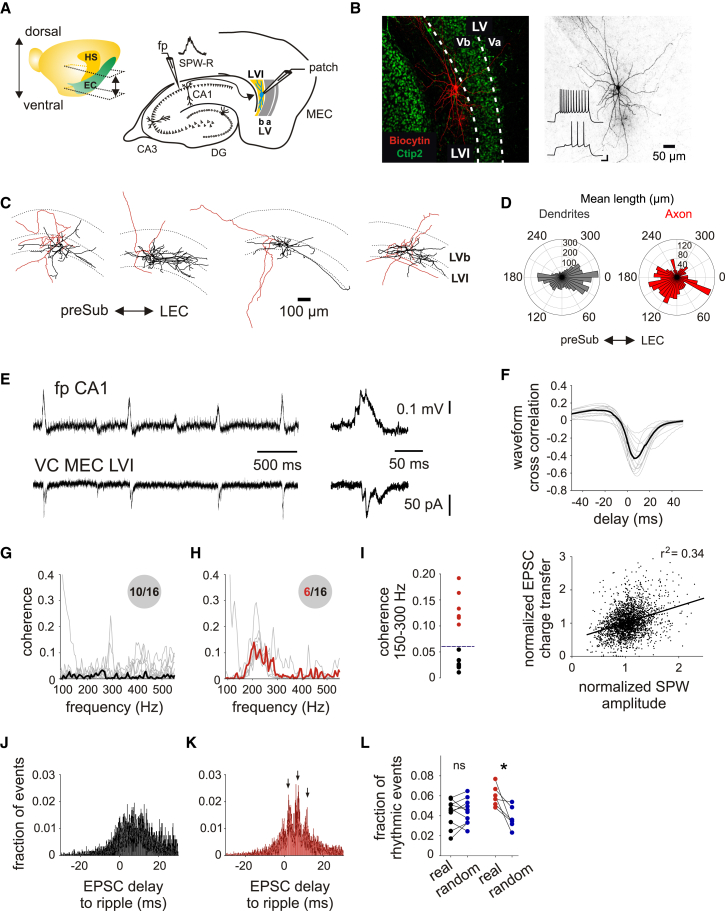
MEC LVI principal cells receive EPSC barrages correlated with SPW-R in CA1 (A) Left: illustration of a mouse brain hemisphere showing the positions of the hippocampus (HS) and EC with the approximate range of horizontal sections used in experiments indicated by dashed lines and arrows. Right: schematic drawing of a horizontal hippocampal–EC slice showing the positioning of field potential (fp) and patch-clamp electrodes. (B) Maximum intensity projection confocal image of a representative horizontal slice showing a recorded LVI principal neuron labeled with biocytin, overlaid with Ctip2 labeling. The right image shows the same neuron in black and white contrast. Both images have equal calibration, scale bar indicated on the right. Insets show firing behavior of the cell in response to 0.25 and 0.35 nA current injections, scale bars correspond to 20 mV and 100 ms. (C) Reconstructions of four representative MEC LVI principal neurons labeled during the recordings illustrated in A with dendrites shown in black and the axon in red. preSub, presubiculum; LEC, lateral entorhinal cortex. (D) Dendritic and axonal mean polar histograms of representative LVI neurons (6 cells from 3 mice). (E) Example traces from a field recording in CA1 (top) and a simultaneous whole-cell voltage-clamp recording from an MEC layer VI principal cell (bottom). Traces on the right show the first pair of events from the left at an expanded timescale. Note the short propagation delay. (F) Top: waveform cross correlations between field and whole-cell recordings from 16 experiments (gray lines), median correlation is shown in black. Bottom: pooled data from 16 simultaneous field and whole-cell voltage-clamp recordings reveal a weak correlation between SPW amplitude and EPSC charge transfer (16 cells from 11 mice). (G) Median (black) of coherence plots (gray) from 10 out of 16 recordings that did not show coherent oscillations in the ripple frequency domain (150–300 Hz) between field potentials during SPW-R in CA1 and EPSC trains in MEC LVI (10 cells from 10 mice). (H) Median (red) of coherence plots (gray) from the remaining 6 out of 16 recordings that showed a prominent peak in coherence at 150–300 Hz between ripple and EPSC oscillations in CA1 and MEC LVI, respectively (6 cells from 5 mice). (I) Summary graph for the quantification of peak coherence between 150 and 300 Hz for all 16 recordings. The ten recordings from G are shown in black and the six recordings from H in red. The dashed line indicates the coherence threshold for classification (see STAR Methods). (J) Median event cross correlation plot for the same recordings as in G. Timepoints of detected ripple and EPSC events were used to compute the delays between each ripple and EPSC event per detected SPW-R in the field trace as a reference. (K) Same as in J for the six recordings presented in H. Note the large peak at 8 ms delay, flanked by two secondary peaks (+/− 5 ms, all labeled by arrows), suggesting rhythmic (∼200 Hz) entrainment of MEC LVI EPSCs by ripple oscillations in CA1 in these cells. (L) The fraction of rhythmic events was computed for 32 event cross correlations. Each of the 16 experiments was analyzed using either real EPSC time points or randomized time points using the same number of EPSC events from MEC LVI neurons per detected SPW-R in CA1. Black dots represent the ten experiments underlying J, red dots represent the six experiments underlying K, blue dots represent matching randomized data. Quantifications of real vs. randomized data were compared using the Wilcoxon signed-rank test, ns *p* = 0.7695, ∗*p* = 0.0313. See also Table S1.

We recorded spontaneously occurring hippocampal SPW-R in stratum pyramidale of area CA1 in mouse horizontal brain slices, comprising the ventral to intermediate hippocampus (Figure 1A). These signals share many properties of naturally occurring SPW-R *in vivo* and have been widely used as an *in vitro* model for this network pattern.^22^^,^^23^^,^^24^ Simultaneously, we recorded excitatory postsynaptic currents (EPSCs) from glutamatergic MEC LVI neurons by holding the cells at the equilibrium potential of GABA_A_R-mediated currents (approximately −90 mV, Figure 1A). Principal neurons in LVI had large round cell bodies with multipolar dendrites that traveled either horizontally within LVI or extended toward the subiculum (Figures 1B–1D). The cells lacked a clearly discernible apical dendrite (Figures 1B–1D) and exhibited a distinct delayed firing pattern without a sag (Figure 1B; Table S1), consistent with previous reports in rats^16^ and mice.^20^ SPW-R propagated efficiently to LVI principal neurons, causing compound EPSCs that were temporally correlated to CA1 sharp waves in all 16 recorded cells (median: EPSC to SPW-R coupling 69%, delay 7.2 ms, *n* = 16; Figures 1E and 1F, top). Sharp wave amplitudes weakly correlated with EPSC charge transfer (r^2^ = 0.34, *n* = 16; Figure 1F, bottom). To investigate whether the fine structure of rhythmic ripple oscillations is transmitted to LVI and preserved within EPSC trains, we quantified the coherence between CA1 field oscillations and LVI EPSCs. This analysis revealed that a subset (6/16) of recorded neurons showed coherent oscillations between hippocampal SPW-R and postsynaptic currents in LVI neurons in the ripple frequency domain (150–300 Hz), while the remaining 10 out of 16 cells did not show increased coherence in this frequency range (Figures 1G–1I). Data from the ten cells without ripple coherence (Figure 1G) exhibited a single smooth peak with a time delay consistent with the previously measured delay between hippocampal sharp waves and compound postsynaptic currents in entorhinal neurons (Figure 1J). In contrast, the six cells with ripple coherence (Figure 1H) showed rhythmic ripple-to-EPSC delay values in the cross correlation with ∼5 ms intervals (∼200 Hz) between the peaks, suggesting that coherent oscillations were indeed caused by ripple-synchronous synaptic input with consistent temporal alignment between multiple ripple and EPSC events per SPW-R (Figure 1K). To test whether the observed rhythmicity truly depends on the precise temporal alignment between ripple cycles and EPSCs, we quantified the fraction of rhythmic events in the histogram and compared results for real EPSC time points with randomized time points (see STAR Methods for details, Figure 1L). This comparison confirmed that the rhythmic structure for the six cells shown in Figure 1K reflects rhythmically modulated EPSCs, while the temporal structure of EPSCs in the other cells (Figure 1J) is indistinguishable from a randomized distribution. Overall, these results indicate that at ventral/intermediate levels of the hippocampus spontaneously occurring hippocampal output signals reliably propagate to MEC LVI, in some cases preserving the high-frequency temporal structure of ripple oscillations.

### Organization of hippocampal projections to MEC layer VI along the dorsoventral axis

**Figure 2:**
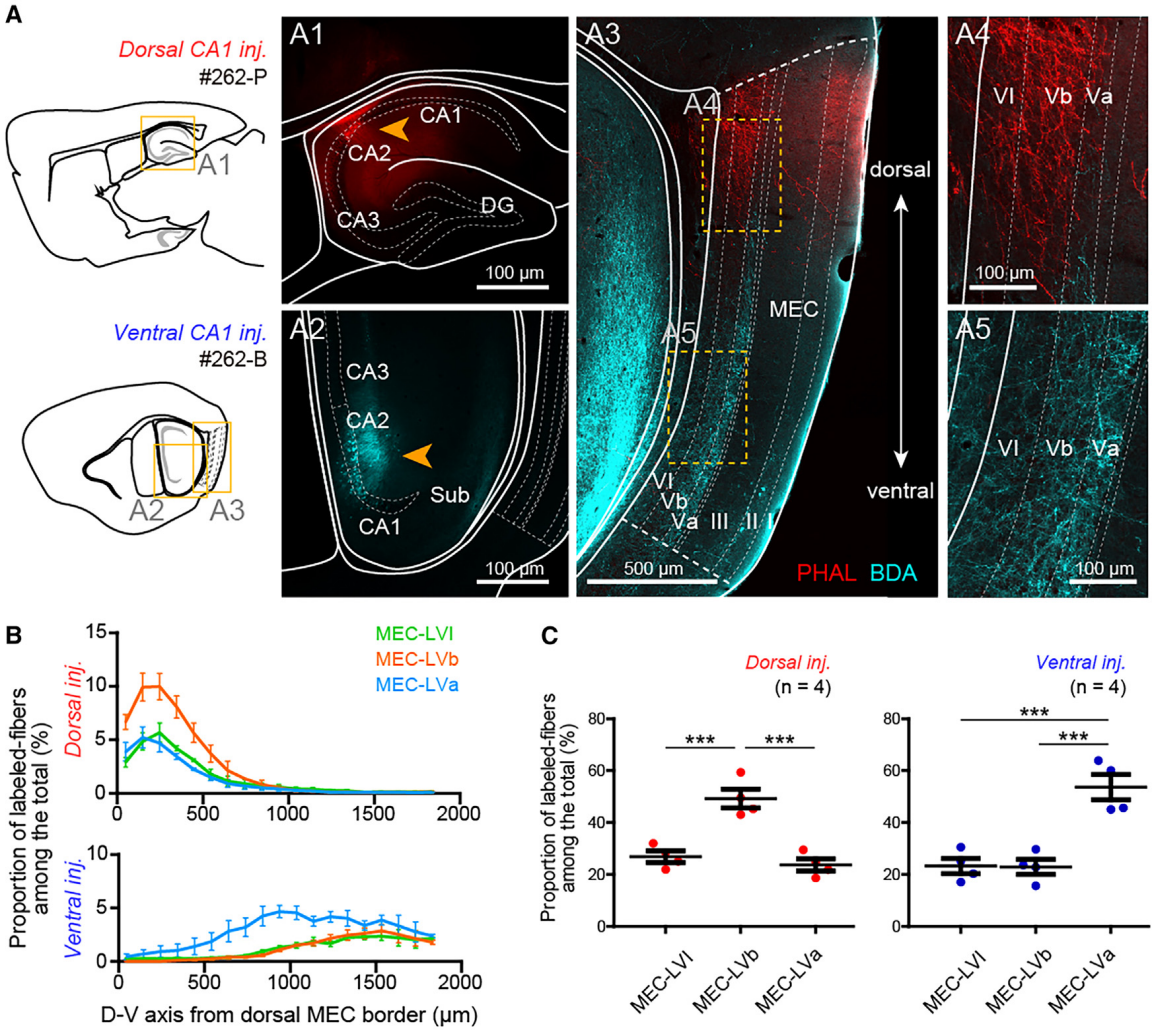
The dorsal and ventral hippocampus target MEC layer VI topographically along the dorsoventral axis (A) Representative sample with a PHA-L injection in dorsal CA1 (case #262-P) and BDA injection in ventral CA1 (case #262-B). The injection sites are shown in A1–A2 (orange arrowhead) and the distribution of labeled axons in MEC is shown in A3–A5. (B) Proportion of labeled fibers in layers VI, Vb, and Va among all labeled fibers along the dorsoventral axis of MEC for samples injected into the dorsal (top, 4 mice) and ventral hippocampus (bottom, 4 mice). (C) Quantification of labeling intensity in MEC LVI, LVb and LVa relative to all labeled fibers for samples injected into the dorsal (red, 4 mice; one-way ANOVA, F_(2,9)_ = 25.3, *p* < 0.001, Bonferroni’s multiple comparison test, ∗∗∗*p* < 0.001) and ventral hippocampus (blue, 4 mice; one-way ANOVA, F_(2,9)_ = 23.03, *p* < 0.001, Bonferroni’s multiple comparison test, ∗∗∗*p* < 0.001). Data are presented as mean ± SEM.

We have previously shown that hippocampal projections to MEC LV differ markedly along the dorsoventral axis.^14^ In order to investigate how dorsal and ventral hippocampal levels interact with MEC LVI circuits, we examined the organization of hippocampal-MEC LVI projections along the dorsoventral axis. We first characterized the projection patterns anatomically by re-analyzing previously generated histological material.^14^ In these samples, anterograde tracers were injected into the output structure of the hippocampal formation, either CA1 or subiculum, at different dorsoventral levels (*n* = 8, Figure 2A, injection sites are shown in Ohara et al;^14^). The distribution of labeled fibers in the deep layers of MEC was subsequently examined in the sagittal plane (Figure 2A). Projections to MEC LVI were organized topographically along the dorsoventral axis: the dorsal hippocampus projected to dorsal LVI, whereas the ventral hippocampus projected to ventral LVI (Figures 2A and 2B). Projections to LVI thus mirror the topographically organized hippocampal projections to MEC LVb, and differ from the innervation pattern of LVa, which is targeted by the ventral hippocampus throughout the dorsoventral MEC axis (Figure 2B). We quantified the innervation density by measuring the proportion of axonal labeling intensity in each layer. Following ventral hippocampal injections, axonal labeling in MEC LVI was comparably strong to LVb, while the proportion of labeled axons in LVa was significantly higher (*p* < 0.001 for LVI vs. LVa and LVb vs. LVa, one-way ANOVA followed by Bonferroni’s multiple comparison test; Figure 2C, right). In contrast, following dorsal injections, axonal labeling in LVI was significantly weaker than in LVb, being comparable to LVa (*p* < 0.001 for LVI vs. LVb and LVb vs. LVa, one-way ANOVA followed by Bonferroni’s multiple comparison test; Figure 2C, left). Hippocampal projections to MEC LVI are thus topographically organized, while the strength of hippocampal innervation in both ventral and dorsal LVI appears to be comparable to the more weakly innervated LV sublayer – LVb in the case of ventral and LVa in the case of dorsal hippocampal projections.

### Functional connectivity between the hippocampus and MEC layer VI

**Figure 3:**
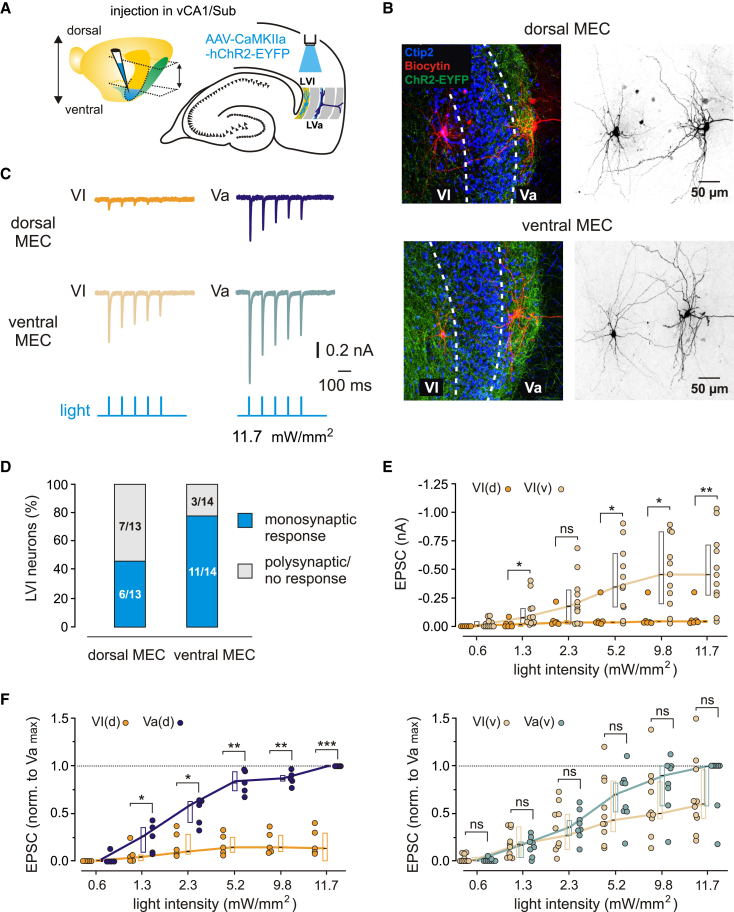
Functional connectivity between the ventral hippocampus and MEC layer VI excitatory neurons (A) Left: illustration of the injection site (blue) in the ventral hippocampus with the approximate range of horizontal sections used in experiments indicated by dashed lines and arrows. Right: schematic drawing of a horizontal hippocampal-EC slice showing the position of light stimulation used to activate the axons of ventral hippocampal neurons infected with AAV-CaMKIIa-hChR2-EYFP. (B) Maximum intensity projection confocal images of representative horizontal slices from the dorsal (top) and ventral MEC (bottom), showing recorded LVI and LVa principal neurons labeled with biocytin, overlaid with Ctip2 labeling and fluorescent staining of hippocampal axons expressing hChR2-EYFP. Right images show the same neurons in black and white contrast. All images have equal calibration, scale bars indicated on the right. (C) Example EPSC traces recorded from LVI and LVa neurons in the same slice in the dorsal (top) and ventral MEC (middle) in response to 1 ms blue light pulses (bottom). (D) Proportion of putative monosynaptic responses from LVI neurons recorded in the dorsal or ventral MEC. (E) Quantification of EPSC amplitudes from LVI neurons recorded in the dorsal (VI(d)) or ventral MEC (VI(v)) in response to light pulses with increasing intensities (VI(d), 6 cells from 4 mice; VI(v), 11 cells from 5 mice). (F) Values from E normalized to the highest LVa response at maximum light intensity (11.7 mW/mm^2^) in each slice in the dorsal (left, VI(d), 5 cells from 4 mice; Va(d), 5 cells from 4 mice) or ventral MEC (right, VI(v), 10 cells from 5 mice: Va(v), 8 cells from 5 mice). All data are presented as median (black line), 25th and 75th percentiles (box). Circles represent individual values. Mann-Whitney U test or one-sample t-test: ∗∗∗*p* < 0.001; ∗∗*p* < 0.01; ∗*p* < 0.05; ns, not significant. See also Figures S1–S3.

**Figure 4:**
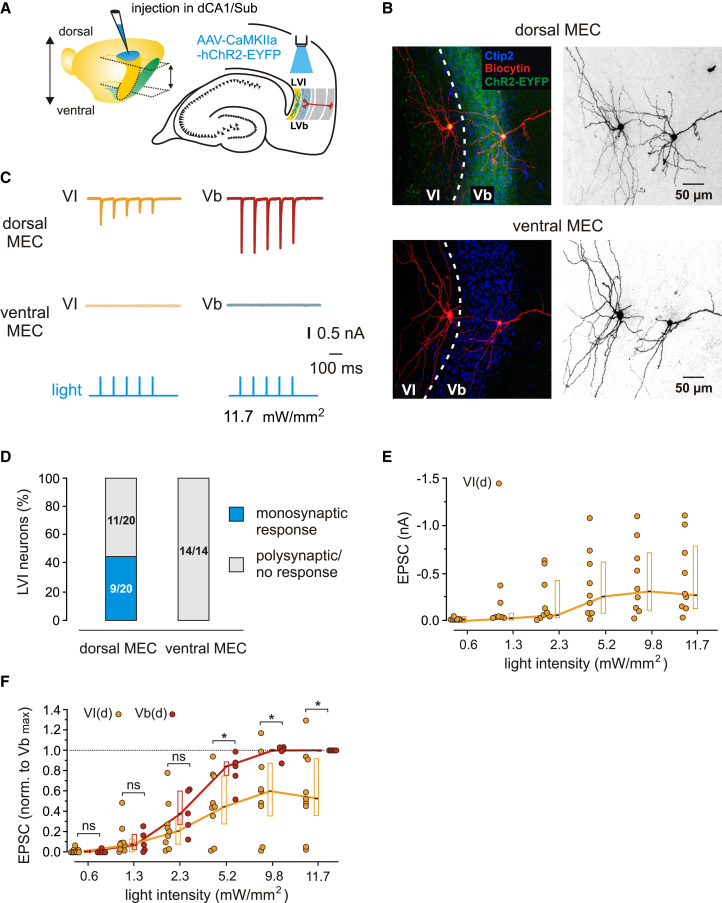
Functional connectivity between the dorsal hippocampus and MEC layer VI excitatory neurons (A) Left: illustration of the injection site (blue) in the dorsal hippocampus with the approximate range of horizontal sections used in experiments indicated by dashed lines and arrows. Right: schematic drawing of a horizontal hippocampal-EC slice showing the position of light stimulation used to activate the axons of dorsal hippocampal neurons infected with AAV-CaMKIIa-hChR2-EYFP. (B) Maximum intensity projection confocal images of representative horizontal slices from the dorsal (top) and ventral MEC (bottom), showing recorded LVI and LVb principal neurons labeled with biocytin, overlaid with Ctip2 labeling and fluorescent staining of hippocampal axons expressing hChR2-EYFP. Right images show the same neurons in black and white contrast. All images have equal calibration, scale bars indicated on the right. (C) Example EPSC traces recorded from LVI and LVb neurons in the same slice in the dorsal (top) and ventral MEC (middle) in response to 1 ms blue light pulses (bottom). (D) Proportion of putative monosynaptic responses from LVI neurons recorded in the dorsal or ventral MEC. (E) Quantification of EPSC amplitudes from LVI neurons recorded in the dorsal MEC in response to light pulses with increasing intensities (9 cells from 4 mice). (F) Values from E normalized to the highest LVb response at maximum light intensity (11.7 mW/mm^2^) in each slice in the dorsal MEC (VI(d), 9 cells from 4 mice; Vb(d), 5 cells from 4 mice). All data are presented as median (black line), 25th and 75th percentiles (box). Circles represent individual values. Mann-Whitney U test or one-sample t-test: ∗*p* < 0.05; ns, not significant. See also Figures S4 and S5.

For a quantitative assessment of the functional connectivity between the hippocampus and MEC LVI neurons, we injected an adeno-associated virus (AAV) expressing hChR2-EYFP under the CaMKII promoter into either ventral or dorsal hippocampal area CA1 (Figures S1 and S4). We then recorded EPSCs from LVI excitatory cells or fast-spiking (FS) interneurons in either the ventral or dorsal MEC, while activating ChR2-expressing hippocampal axons by illuminating LVI with blue light (Figures 3A, 4A, S3A and S5A). For a comparison of innervation efficacy between LVI and LV, we additionally recorded responses from LVa or LVb glutamatergic neurons in all slices. In line with anatomical tracing experiments, injections into ventral CA1 (Figure S1) resulted in a plexus of fluorescently labeled fibers in LVI at ventral MEC levels, while at more dorsal levels labeling in LVI was minimal to absent (Figures 3B and S3B). In the ventral MEC, the majority (11/14) of excitatory LVI neurons reliably responded to light stimulation with short latencies and fast 20%–80% EPSC rise times (11.7 mW/mm^2^, median: amplitude −0.45 nA, latency 2.36 ms, 20%–80% rise time 1.22 ms, *n* = 11; Figures 3C–3E), consistent with monosynaptic innervation by the ventral hippocampus. The remaining cells (3/14) responded with longer latencies (median 8.32 ms), suggesting putative polysynaptic innervation (Figure 3D). The EPSCs were sensitive to the AMPA and kainate receptor antagonist CNQX but not to the GABA_A_ receptor antagonist gabazine (Figure S2A). Furthermore, while the EPSCs were blocked by CNQX at a negative holding potential (−70 mV), NMDA receptor currents could still be recorded at a positive holding potential (+40 mV, Figure S2A). This finding confirms the monosynaptic nature of the EPSCs, as polysynaptic activity would be blocked by CNQX.^20^ Notably, in the dorsal half of MEC, only 6/13 LVI neurons responded to light stimulation after expression of hChR2 in the ventral hippocampus, with the responding cells displaying minimal EPSC amplitudes (11.7 mW/mm^2^, median: amplitude −45 pA, latency 2.36 ms, 20–80% rise time 1.04 ms, *n* = 6; *p* = 0.0057 for median LVI dorsal vs. LVI ventral amplitude, Mann-Whitney test; Figures 3C–3E).

To compare the strength of LVI innervation by the ventral hippocampus with LV, we normalized the responses of LVI neurons to the highest LVa EPSC amplitude at maximum light intensity in each slice, as excitatory LVa cells were previously shown to receive strong putative monosynaptic input from the ventral hippocampus.^14^ Normalized short-latency EPSC amplitudes in LVI cells in the ventral half of MEC tended to be lower than amplitudes measured in LVa neurons (11.7 mW/mm^2^, median: LVa, 1.00, *n* = 8; LVI, 0.60, *n* = 10; Figure 3F, right), although this difference did not reach significance (*p* = 0.2577, Mann-Whitney test). Normalized short-latency EPSC amplitudes in LVI neurons in the dorsal half of MEC, in contrast, were substantially lower than LVa responses, reaching an over 7-fold difference at higher stimulation intensities (11.7 mW/mm^2^, median: LVa, 1.00, *n* = 5; LVI, 0.13, *n* = 5; *p* = 0.0002, one-sample t-test; Figure 3F, left). We further investigated whether the ventral hippocampus also targets LVI FS interneurons. In general, innervation patterns for FS cells in both the ventral and dorsal MEC were comparable to excitatory neurons. In the ventral MEC, light stimulation evoked short latency (median 1.97 ms) EPSCs in 6/8 FS cells (Figures S3C and S3D), with the EPSC amplitudes comparable to those recorded from principal cells (11.7 mW/mm^2^, median −0.41 nA, *n* = 6; Figure S3E). In contrast, in the dorsal MEC, only half (4/8) of the recorded FS cells responded to light stimulation (Figures S3C and S3D), displaying small amplitudes similarly to principal cells (11.7 mW/mm^2^, median: amplitude −86 pA, latency 2.11 ms, *n* = 4; Figure S3E). Together, these results corroborate our findings from the anatomical tracing experiments, showing that the ventral hippocampus innervates LVI excitatory and FS interneurons in the ventral MEC.

We next recorded from LVI excitatory or FS interneurons in both the ventral and dorsal MEC following injections of the AAV-CaMKII-hChR2-EYFP virus into dorsal hippocampal area CA1 (Figure S4). Consistently with anatomical tracing experiments, dorsal CA1 injections resulted in weak to moderate axonal fluorescence in LVI in the dorsal half of MEC, while none of the injections displayed fluorescent labeling in the ventral MEC (Figures 4B and S5B). The lack of projections from the dorsal hippocampus to ventral MEC LVI was confirmed by recordings from a total of 14 LVI excitatory and 6 FS interneurons, all of which failed to respond to light stimulation (Figures 4C and 4D, and S5C-S5E). In the dorsal MEC, roughly half (9/20) of excitatory LVI neurons responded to light stimulation with short latencies and fast EPSC rise times (11.7 mW/mm^2^, median: amplitude −0.29 nA, latency 2.27 ms, 20%–80% rise time 0.78 ms, *n* = 9; Figures 4C–4E), indicating putative monosynaptic innervation by the dorsal hippocampus. Of the remaining 11 cells, two failed to show any discernible response and the other nine responded with longer latencies (median 6.52 ms), suggesting putative polysynaptic input to these cells (Figure 4D). We again compared the strength of LVI innervation by the dorsal hippocampus with LV by normalizing the responses of LVI neurons to the highest LVb EPSC amplitude at maximum light intensity in each slice, as excitatory LVb cells have been shown to receive strong putative monosynaptic input from the dorsal hippocampus.^13^^,^^14^^,^^25^ Normalized EPSC amplitudes in short-latency LVI cells in the dorsal half of MEC reached roughly half of the amplitude values measured in LVb neurons (11.7 mW/mm^2^, median: LVb, 1.00, *n* = 5; LVI, 0.52, *n* = 9; *p* = 0.0160, one-sample t-test; Figure 4F). Finally, we examined the innervation of LVI FS interneurons in the dorsal MEC by the dorsal hippocampus. In total, 12/15 FS cells responded to light stimulation with short latencies (median 1.85 ms, Figures S5C and S5D), demonstrating a higher ratio of short-to long-latency/non-responding cells compared to excitatory neurons. Nevertheless, short-latency current amplitudes in FS interneurons were comparable to those measured in principle cells (11.7 mW/mm^2^, median −0.32 nA, *n* = 12; Figure S5E). In summary, dorsal hippocampal outputs to MEC LVI are confined to the dorsal MEC, where they innervate both FS interneurons and LVI principal cells more weakly than LVb principal cells.

### Local connectivity within MEC layer VI and interlaminar connectivity between layers VI and Vb

**Figure 5:**
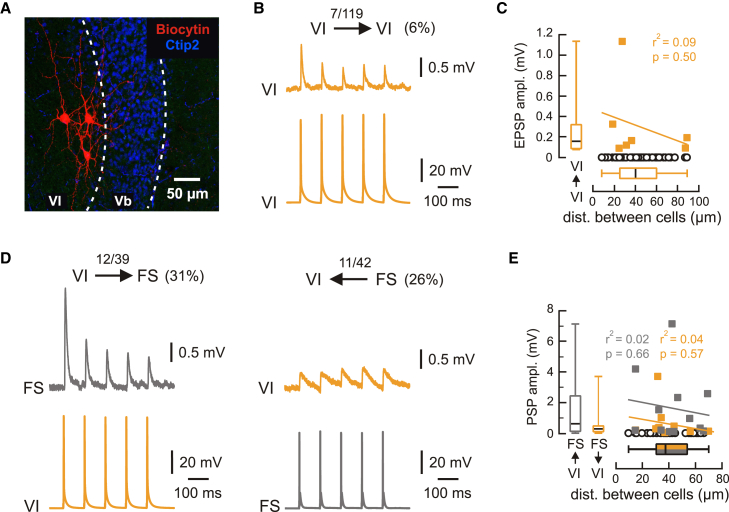
Local connectivity within MEC layer VI (A) Maximum intensity projection confocal image of a typical horizontal slice showing recorded MEC LVI principal neurons labeled with biocytin, overlaid with Ctip2 staining. (B) Example traces from a paired recording between connected LVI excitatory neurons, showing presynaptic APs and associated PSPs. (C) PSP amplitudes from all tested VI-to-VI pairs relative to the distance between recorded neurons. Yellow squares represent connected and open black circles non-connected pairs (note the large number of non-connected pairs). The boxplot for PSP amplitudes and the correlation analysis represent data from connected pairs only, while the boxplot for distances represents all recorded cells. (D) Example traces from paired recordings between connected LVI excitatory and FS interneurons. (E) Same analysis as in C for all tested VI-to-FS (connected pairs in gray) and FS-to-VI pairs (connected pairs in yellow). See also Figure S6 and Table S2.

Since both the ventral and dorsal hippocampus target excitatory and inhibitory neurons in MEC LVI, we next asked how the incoming signals are processed within MEC deep layers. We performed a series of paired recordings to determine the connectivity scheme between different combinations of LVI and LV excitatory and FS interneurons. We first tested local connectivity within LVI. Functional connections between LVI excitatory neurons were relatively sparse (5.9%, 7 of 119 pairs; Figures 5A–5C). The detected connections were exclusively depressing, with an approximately 50% amplitude decrease between the first and the second postsynaptic potential (PSP; Figure S6A, top). In comparison, connections between LVI excitatory and FS interneurons were much more frequent in both directions: while connectivity from LVI excitatory cells to local FS interneurons was 30.8% (12 of 39 pairs, Figure 5D, left, 5E, and S6, middle), connectivity from FS to LVI excitatory cells was 26.2% (11 of 42 pairs, Figure 5D, right, 5E, and S6A, bottom). All observed connection frequencies and nearly all measured PSP parameters were comparable between the dorsal and ventral MEC (Table S2).

**Figure 6:**
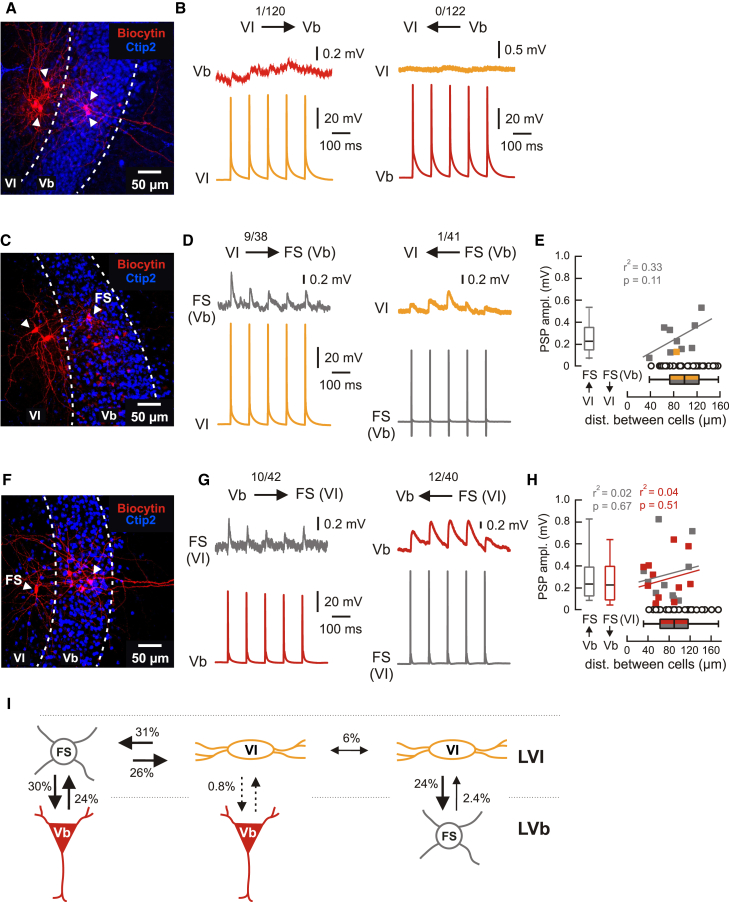
Interlaminar connectivity between MEC layers VI and Vb (A) Maximum intensity projection confocal image of a typical horizontal slice showing recorded MEC LVI and LVb principal neurons (white arrowheads) labeled with biocytin, overlaid with Ctip2 staining. (B) Example traces from paired recordings between LVI and LVb excitatory neurons, showing presynaptic APs and, for the single VI-to-Vb connection, the associated PSPs. (C) Maximum intensity projection confocal image showing recorded LVI principal cells and a LVb FS interneuron labeled with biocytin, overlaid with Ctip2 staining. The LVI cell indicated by the white arrowhead was connected to the FS interneuron. (D) Example traces from paired recordings between connected LVI excitatory and LVb FS interneurons. (E) PSP amplitudes from all tested pairs relative to the distance between recorded neurons. Gray squares represent connected VI-to-FS (Vb) pairs, the yellow square the single connected FS (Vb)-to-VI pair, and open black circles non-connected pairs (note the large number of non-connected pairs). The boxplot for PSP amplitudes and the correlation analysis represent data from connected pairs only, while the boxplot for distances represents all recorded cells. (F) Maximum intensity projection confocal image showing a recorded LVI FS interneuron and LVb principal cells labeled with biocytin, overlaid with Ctip2 staining. The LVb cell indicated by the white arrowhead was reciprocally connected to the FS interneuron. (G) Example traces from paired recordings between connected LVb excitatory and LVI FS interneurons. (H) Same analysis as in E for all tested Vb-to-FS (VI) (connected pairs in gray) and FS (VI)-to-Vb pairs (connected pairs in red). (I) Schematic representation of intra- and interlaminar connectivity between LVI and LVb excitatory and FS interneurons. See also Figure S6 and Table S3.

MEC LVb cells have been shown to receive putative monosynaptic input from both the dorsal and ventral hippocampus and to project locally within MEC to layers III and II.^11^^,^^12^ To investigate whether signals arriving in LVI are passed on to LVb and thus potentially modulate information flow to superficial layers, we next tested connectivity between layers VI and Vb. Direct connections between LVI and LVb excitatory neurons were exceedingly rare in both directions. We only observed one weak connection from an LVI cell to a LVb neuron (1 of 120 pairs, Figures 6A and 6B, left) and failed to find any connections from LVb to LVI cells (0 of 122 pairs, Figures 6A and 6B, right). As before, connections between excitatory cells and FS interneurons were significantly more frequent than connections between principal neurons. Connectivity from LVI principal cells to LVb FS interneurons was 23.7% (9 of 38 pairs, Figures 6C and 6D, left, and 6E), whereas LVb principal cells were connected to LVI FS interneurons in 23.8% of tested pairs (10 of 42 pairs, Figures 6F and 6G, left, and 6H). While connections from LVI excitatory to LVb FS cells were expectedly depressing (Figure S6B, top), connections from LVb excitatory to LVI FS cells were in many cases facilitating (Figure S6B, middle).

Connectivity from LVI FS interneurons to LVb excitatory cells was 30% (12 of 40 pairs, Figures 6F and 6G, right, 6H, and S6B, bottom). As a notable exception, connections from LVb FS interneurons to LVI principal cells were almost absent (1 of 41 pairs, Figures 6C and 6D, right, and 6E), suggesting asymmetric inhibition of the neighboring layer by LVI and LVb FS interneurons. Similarly to local connectivity within LVI, all observed connection frequencies and most PSP parameters were comparable between the dorsal and ventral MEC (Table S3). In summary, we found that connections between principal cells within LVI are fairly rare and interlaminar connections between LVI and LVb principal neurons are almost absent. The low connectivity between principal neurons is contrasted by considerably higher connectivity between excitatory cells and FS interneurons both within LVI as well as between layers VI and Vb (Figure 6I).

### Interlaminar connectivity between layers VI and Va

**Figure 7:**
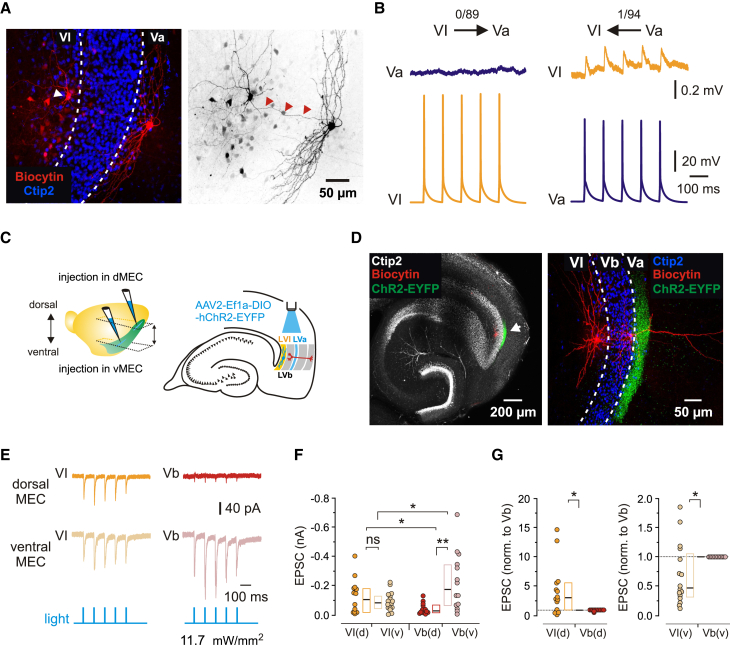
Interlaminar connectivity between MEC layers VI and Va (A) Maximum intensity projection confocal image of a typical horizontal slice showing a recorded MEC LVI (white arrowhead) and a LVa principal neuron labeled with biocytin, overlaid with Ctip2 staining. The right image shows the same neurons in black and white contrast, red arrowheads indicate the axon of the LVa cell. Both images have equal calibration, scale bar indicated on the right. (B) Example traces from paired recordings between LVI and LVa excitatory neurons showing presynaptic APs and, for the single Va-to-VI connection, the associated PSPs. (C) Left: illustration of the AAV2-Ef1a-DIO-hChR2-EYFP injection site (blue) in dorsal or ventral MEC LVa in Rbp4-Cre mice. Right: schematic drawing of a horizontal hippocampal-EC slice showing the position of light stimulation used to activate the axons of LVa neurons expressing hChR2-EYFP. (D) Left: low magnification confocal image of a representative horizontal slice from a virus-injected Rbp4-Cre mouse showing the specific expression of hChR2-EYFP in MEC LVa. Right: maximum intensity projection confocal image from the indicated region in the left image showing a recorded MEC LVI and a LVb principal neuron labeled with biocytin, overlaid with Ctip2 labeling and hChR2-EYFP staining. (E) Example EPSC traces recorded from LVI and LVb neurons in the dorsal (top) and ventral MEC (middle) in response to 1 ms blue light pulses (bottom). (F) Quantification of EPSC amplitudes from LVI and LVb neurons in the dorsal (d) or ventral MEC (v) in response to maximum intensity light pulses (11.7 mW/mm^2^, VI(d), 14 cells from 7 mice; VI(v), 16 cells from 6 mice; Vb(d), 12 cells from 7 mice; Vb(v), 14 cells from 6 mice). (G) Values from F normalized to the highest LVb response in each slice in the dorsal (left, VI(d), 14 cells from 7 mice; Vb(d), 12 cells from 7 mice) or ventral MEC (right, VI(v), 16 cells from 6 mice; Vb(v), 14 cells from 6 mice). All data are presented as median (black line), 25th and 75th percentiles (box). Circles represent individual values. Unpaired or one-sample t-test: ∗∗*p* < 0.01; ∗*p* < 0.05; ns, not significant.

Excitatory MEC LVa neurons constitute the main MEC output pathway, relaying signals to various telencephalic structures.^13^ To determine whether LVI neurons are able to influence these MEC output signals and, conversely, whether MEC outputs are copied to LVI, we tested connectivity between excitatory neurons in layers VI and Va. Similarly to the minimal connectivity observed between LVI and LVb principal cells, we only found one connection from a LVa to an LVI excitatory neuron (1 of 94 pairs, Figures 7A and 7B, right) and failed to find any connections from LVI to LVa cells (0 of 89 pairs, Figures 7A and 7B, left). Distances between cells in these recordings approached and occasionally exceeded 200 μm – a distance at which the chance of finding connections may be generally low.^26^ However, with our optimized slicing angle (see STAR Methods) we could regularly follow axons running parallel to the slice surface for significantly longer distances. In fact, LVa axons almost invariably passed through LVI toward the alveus (Figure 7A) and in 82% of tested pairs we observed at least one LVa axon collateral passing within 50 μm of the soma of the recorded LVI cell, with a median distance of 25 μm (*n* = 94). It is therefore likely that strong connectivity between LVa and LVI principal neurons would have been detected with our methods. Nevertheless, we further characterized LVa to LVI projection patterns at the population level using Rbp4-Cre mice, which in MEC express Cre specifically in LVa principal cells.^27^ In these mice, we injected a Cre-dependent AAV expressing hChR2-EYFP into either ventral or dorsal MEC LVa and subsequently recorded light-induced EPSCs from LVI excitatory neurons in either the ventral or dorsal MEC (Figure 7C). For comparison, in each slice we additionally recorded EPSCs from LVb excitatory neurons. In both the ventral and dorsal MEC, the viral injections resulted in strong fluorescent labeling in LVa (Figure 7D). When activated optogenetically, all LVI neurons reliably responded to light stimulation with short latencies (11.7 mW/mm^2^, median: 2.15 ms, *n* = 30; LVI ventral, 2.26 ms, *n* = 16; LVI dorsal, 2.10 ms, *n* = 14) and fast 20%–80% EPSC rise times (11.7 mW/mm^2^, median: 1.07 ms, *n* = 30; LVI ventral, 1.11 ms, *n* = 16; LVI dorsal, 1.05 ms, *n* = 14; Figure 7E), comparable with responses recorded from LVI neurons after CA1 injections. Amplitudes of evoked EPSCs in LVI cells, however, were relatively small (11.7 mW/mm^2^, median −88 pA, *n* = 30) and did not differ significantly between the two MEC halves (11.7 mW/mm^2^, median: LVI ventral, −88 pA, *n* = 16; LVI dorsal, −110 pA, *n* = 14; *p* = 0.3719, unpaired t-test; Figure 7F). All LVb neurons also responded to the stimulation of hChR2-expressing LVa axons with short latencies (11.7 mW/mm^2^, median: 2.21 ms, *n* = 26; LVb ventral, 2.18 ms, *n* = 14; LVb dorsal, 2.32 ms, *n* = 12) and fast EPSC rise times (11.7 mW/mm^2^, median: 0.79 ms, *n* = 26; LVb ventral, 0.80 ms, *n* = 14; LVb dorsal, 0.78 ms, *n* = 12; Figure 7E). Furthermore, LVb EPSC amplitudes measured across the full MEC dorsoventral axis (11.7 mW/mm^2^, median −71 pA, *n* = 26) were comparable to responses measured from LVI neurons (*p* = 0.9411, Mann-Whitney test). In striking contrast to LVI responses, however, LVb EPSC amplitudes exhibited a major difference between the ventral and dorsal MEC. While LVb amplitudes in the ventral MEC were stronger than LVI responses (11.7 mW/mm^2^, median: LVb ventral, −176 pA, *n* = 14; LVI ventral, −88 pA, *n* = 16; *p* = 0.0170, unpaired t-test; Figure 7F), LVb amplitudes in the dorsal MEC were minimal (11.7 mW/mm^2^, median: LVb dorsal, −31 pA, *n* = 12; LVI dorsal, −110 pA, *n* = 14; *p* = 0.0361, unpaired t-test; Figure 7F). This difference remained unchanged after LVI responses were normalized to the highest LVb current amplitude in each slice to account for differences in fluorescence intensity between injections (11.7 mW/mm^2^, median: LVb ventral, 1.00, *n* = 14; LVI ventral, 0.47, *n* = 16; *p* = 0.0306, one-sample t-test; LVb dorsal, 1.00, *n* = 12; LVI dorsal, 3.05, *n* = 14; *p* = 0.0130, one-sample t-test; Figure 7G). Together, these results suggest that MEC outputs mediated by LVa neurons might be partially copied to LVI principal cells through limited excitatory inputs, whereas innervation in the opposite direction is likely weak to minimal.

## Discussion

The functional organization of MEC deep layers is gaining increasing interest due to their distinct layer-specific input-output connectivity, suggesting divergent processing of hippocampal outputs. Here, we provide an in-depth analysis of MEC layer VI, a hitherto understudied part of the entorhinal network. Our electrophysiological data show that LVI receives patterned network activity from the hippocampus, similarly to layer V. Using a combination of optogenetic and anatomical circuit tracing, we confirm direct hippocampal projections to LVI and demonstrate that these are topographically organized along the dorsoventral axis. Finally, our paired recordings indicate that excitatory connections between neurons in layers VI and V are very sparse in both directions, suggesting that layer VI forms a largely independent subnetwork for the processing of hippocampal information. Together, these results establish principles of signal flow in the hippocampal-MEC LVI output circuit and in the MEC deep layer network.

### Propagation of SPW-R to MEC LVI

We report that hippocampal SPW-R reliably propagate to MEC LVI. The majority of SPW-R (69%) elicited EPSCs in recorded LVI neurons, which is comparable to our previous data for LVa cells (76%) but higher than for LVb neurons (42%).^15^ Sharp waves are superimposed by very fast network oscillations (ripples) that entrain discharges of hippocampal pyramidal cells.^23^^,^^28^ In a number of LVI cells (38%), EPSC trains evoked by SPW-R showed precise temporal alignment with individual ripple cycles in area CA1, indicating that during the transfer of ripple activity to LVI the temporal precision of this network pattern is maintained. The temporal entrainment of LVI neurons by ripples is in contrast to our previous observations in LV.^29^ This may either be caused by differences in connectivity and local network structure or by better preservation of connections during slice preparation due to the closer proximity of LVI to the hippocampus. Overall, our *ex vivo* data on the propagation of rhythmic activity patterns to LVI align with previous *in vivo* recordings where the discharge of entorhinal deep layer neurons was found to be synchronized to a 200 Hz oscillation during SPW-R events.^30^^,^^31^ It was recently shown that a subset of MEC LV/VI cells are locked to hippocampal theta-band oscillations during periods of activity. The same cells were preferentially coordinated with hippocampal replay events during periods of rest or sleep, indicating that they support memory formation and consolidation in hippocampal-entorhinal networks.^10^ Our *ex vivo* SPW-R recordings in slices may reflect the underlying functional connectivity between the hippocampus and MEC deep layers, including hard-wired connections and, potentially, remnants of previously established experience-dependent connectivity weights. In this case, the subset of neurons showing ripple-entrainment of SPW-R-induced EPSCs *in vitro* may be part of the experience-dependent theta-modulated cells.^10^ Layer VI neurons provide direct back-projections to all hippocampal subregions.^20^ Our present results therefore suggest that LVI is part of a hippocampal-MEC-hippocampal loop, which is active during SPW-R, enabling reverberating activity patterns. Interestingly, both LVa and LVb neurons are similarly able to transfer hippocampal output signals back to the hippocampus, either directly or by indirect pathways. While LVb neurons strongly innervate CA1-projecting MEC LIII neurons,^11^^,^^12^ neocortically projecting LVa neurons copy their outputs directly to CA1 pyramidal cells and interneurons.^27^ Together, these feedback inputs may constitute an important feature of MEC deep layers, allowing precise coordination of hippocampal-cortical interactions during memory formation and consolidation.

### Dorsoventral organization of the hippocampal projection to MEC LVI

The propagation of SPW-R to LVI suggests direct functional connections between hippocampal output regions and LVI neurons. Indeed, several studies have found that neurons in area CA1 and the subiculum project to LVI.^4^^,^^5^^,^^6^^,^^20^^,^^21^ We confirmed a putative monosynaptic projection from the hippocampus to MEC LVI by optogenetically activating hippocampal axons while directly recording from LVI neurons. The connections were topographically organized along the dorsoventral MEC axis, mirroring the distribution of hippocampal projections to LVb and differing from projections to LVa, which is targeted along the entire dorsoventral MEC axis by the ventral hippocampus.^14^ Compared to LV neurons, however, cells in LVI received weaker hippocampal input. First, the percentage of LVI neurons responding with a short latency in the dorsal MEC was lower than the percentage of short-latency LVb cells. Second, EPSC amplitudes in LVI neurons were smaller than in LVb cells in the dorsal and tended to be smaller than in LVa cells in the ventral MEC. The relatively weak activation of LVI neurons in the dorsal MEC by hippocampal output signals may give more weight to other projections onto LVI neurons, including projections from the lateral EC,^32^ which would support the integration of spatial information and contextual contents.^33^^,^^34^ The idea of convergent input has previously been suggested for LVa neurons in the dorsal MEC. Dorsal LVa cells are weakly innervated by the dorsal hippocampus,^13^^,^^15^ but receive auxiliary input from the ventral hippocampus,^14^ potentially enabling the integration of spatial and affective memory signals.^35^

### Local and interlaminar connectivity of LVI neurons

Our paired recordings revealed higher functional connectivity between principal neurons within layer LVI, compared to sparse interlaminar connectivity between LVI and LV principal cells. Connectivity between LVI principal neurons was ∼6%, which is almost identical to the previously reported value for MEC LVb neurons,^15^ but markedly lower than the recurrent connectivity between principal neurons in MEC LVa (∼16%; Rozov et al.^15^). Similarly low connectivity values were also found for principal neurons in MEC LIII.^36^ Recurrent connectivity is thus not prominent in LVI, indicative of a relay function in a feedforward network structure, as opposed to local signal amplification in recurrent networks like CA3,^37^^,^^38^ or MEC LVa.^15^ We also assessed connectivity between excitatory (principal) neurons and FS inhibitory interneurons within LVI. Our data revealed relatively common synaptic connections in both directions (∼30%), suggesting strong excitatory-inhibitory loops, which may give rise to local network oscillations.^24^^,^^39^^,^^40^ Indeed, high-frequency oscillations in the ripple^30^^,^^31^ and gamma^41^ frequency bands have been observed in the deep layers of MEC of behaving rodents *in vivo*.

In contrast, our paired recordings demonstrate only minimal connectivity between principal cells in LVI and LVb or LVI and LVa in both directions. We cannot exclude that a complex spatial organization of connections might exist between some of these neurons that was not fully preserved during slice preparation or that was not taken into account during the sampling of neurons. Together with the larger distance between pairs of neurons in these recordings, this might have led to an underestimation of actual connectivity. Regardless, our results are in agreement with previous tracing studies that have similarly found sparse to minimal bidirectional projections between MEC layers VI and Vb,^20^ or VI and Va.^20^^,^^42^ Moreover, the target cells of the projection from LVa to LVI appear to be overwhelmingly interneurons, rather than principal cells.^42^ We did observe reliable responses in LVI principal neurons following the activation of LVa axons in Rbp4-Cre mice, which enable specific labeling of LVa principal cells.^27^ However, in these recordings LVI amplitudes across the full dorsoventral MEC axis were comparable to responses measured from LVb principal neurons, which have previously been shown to be sparsely innervated by LVa neurons.^15^ Our data thus indicate that direct excitatory crosstalk between layers VI and V is weak, mirroring the low connectivity between layers Va and Vb.^12^^,^^15^ Interestingly, we found that the innervation of LVb principal cells by LVa neurons differs along the dorsoventral MEC axis, with LVa cells exciting LVb neurons only minimally in the dorsal but more strongly in the ventral MEC. We have previously found an analogous connectivity scheme for projections from LVb to LVa principal cells, which are also minimal in the dorsal and stronger in the ventral MEC.^12^ Our present data thus provide another example of the complex dorsal-ventral gradients in hippocampal and entorhinal connectivity.^7^^,^^13^^,^^14^^,^^15^ Together with previous data, our findings indicate that MEC deep layers process hippocampal output signals largely in parallel, without significant excitatory crosstalk between the different deep layer subnetworks. In addition, our present and previous^15^ findings generally demonstrate markedly higher (20–30%) interlaminar connectivity between excitatory cells and FS interneurons, with the exception of low connectivity from LVb FS to LVI principal neurons (2%). These intense inhibitory interactions would be suited to recruit all deep layer subnetworks into common oscillatory regimes.^43^^,^^44^ They may also serve to suppress alternative pathways following strong activation of principal cells in one of the layers.

### Functional roles of LVI neurons

Recently, the first detailed functional interrogation of LVI revealed that LVI neurons project to all subfields of the hippocampus, providing the strongest excitatory drive onto CA3 pyramidal cells.^20^ In addition, LVI neurons project to the lateral septum and the anterior thalamic nuclei.^19^^,^^20^ Ablation or optical inhibition of the LVI cell population *in vivo* led to impaired spatial information processing and memory formation, confirming the functional relevance of these projections.^20^ It has been suggested that highly orchestrated firing of LVI neurons might contribute to the synchronization of principal neuron spiking activity in the hippocampal network, thereby promoting the stabilization of hippocampal firing patterns.^20^ Based on our data, LVI neurons in this model could be recruited directly by principal cells in area CA1 or the subiculum, while the firing of LVI neurons could be coordinated by the strong interactions between LVI excitatory and FS interneurons. On the other hand, the propagation of SPW-R to LVI provides LVI neurons with activity patterns instrumental in memory consolidation. Interestingly, the anteromedial thalamus was recently shown to coordinate the transfer of salient hippocampal representations to the neocortex for long-term storage, forming a complementary pathway to the neocortical projections originating in EC LVa.^45^ Although the anterior thalamus also receives robust input from the subiculum both directly and through the mammillary bodies,^46^ the projection from LVI neurons might form an alternative pathway through which memory-related activity patterns reach the anterior thalamus.

In summary, our data reveal principles of neuronal connectivity and network integration in the deepest layer of MEC. Layer VI forms a separate input layer for hippocampal signals with distinct properties and downstream connections, compared to layers Va and Vb. These results add to the growing insight into the complex input-output connectivity patterns in EC, which, in addition to the prominent hippocampal back-projections of EC LVI neurons, have recently prompted the discovery of several other non-canonical projections: MEC LVa neurons serve as a network hub, routing direct input from the secondary visual cortex to other MEC neurons and hippocampal CA1.^42^ Butola et al. have demonstrated a new direct hippocampal input to MEC layers II and III, where the recipient neurons integrate hippocampal information with cortical sensory inputs, enabling novelty detection for objects and locations.^47^ The topographical dorsoventral organization of the hippocampal projection to LVI suggests that information conveyed to the LVI network by the hippocampal poles might remain segregated. This contrasts with layer Va, which receives input from the ventral hippocampus throughout its dorsoventral extent, before transmitting the signals to telencephalic structures. Such layer-specific differences in functional architecture set important boundary conditions for context-, state- and emotion-dependent processing of sensory and mnestic information in the hippocampal-entorhinal system.

### Limitations of the study

Paired recordings between neurons in different cortical layers can underestimate actual connectivity if the distances between sampled neurons are large or connections between neurons feature a specific spatial organization that is not preserved during slice preparation or is not accounted for during the sampling of neurons. It is therefore possible that *in vivo* connectivity between layers VI and V is higher than reported in this study. This is particularly relevant for connections from LVI to LVa neurons, as the cells were separated by a considerable distance and connectivity in this direction was not tested at the population level. Second, for a complete understanding of the integration of LVI neurons into the MEC circuitry, it will be important for future studies to determine how LVI cells interact with neurons in superficial MEC layers. Lastly, in this study we focused on direct hippocampal projections to MEC LVI and did not consider indirect pathways through which hippocampal output signals might reach MEC LVI neurons. For example, hippocampal output structures project extensively to LV^14^ and likely LVI^20^ in the lateral EC, from where signals could be routed to MEC LVI through direct intra-entorhinal projections.^32^

## Resource availability

### Lead contact

Further information and requests for resources and reagents should be directed to and will be fulfilled by the lead contact, Alexei V. Egorov (alexei.egorov@urz.uni-heidelberg.de).

### Materials availability

This study did not generate new unique reagents.

### Data and code availability

• Data for the propagation of SPW-R to LVI and for the anatomical tracing of dorsal and ventral hippocampal projections have been uploaded to the heiDATA repository and are publicly available at https://doi.org/10.11588/data/EDAJD7.
• This paper does not report original code.
• All remaining data and any additional information required to reanalyze the data reported in this paper is available from the lead contact upon request.

## Acknowledgments

This work was supported by the following grants and organizations: the 10.13039/501100001659Deutsche Forschungsgemeinschaft (DFG, German Research Foundation) No. 430282670 (EG134/2-1) to A.V.E.; PRESTO from the 10.13039/501100002241Japan Science and Technology Agency (JPMJPR21S3) to S.O. Further support (to F.C.R.) was provided by the 10.13039/501100005416Research Council of Norway (315935). We thank the Nikon Imaging Center at Heidelberg University for access to the Nikon AX laser scanning confocal microscope.

## Author contributions

M.R., A.D., and A.V.E. conceptualized the study and wrote the manuscript with input from S.O. and F.C.R. M.R. carried out whole-cell recordings. S.O. collected and analyzed anatomical data. J.W. performed SPW-R recordings and F.C.R. analyzed SPW-R data. M.R., J.W., and A.V.E. performed the electrophysiological and corresponding morphological data analysis.

## Declaration of interests

The authors declare no competing interests.

## STAR★Methods

### Key resources table

**Table:** 

REAGENT or RESOURCE	SOURCE	IDENTIFIER
**Antibodies**		

Rat anti-Ctip2	Abcam	Cat# ab18465; RRID:AB_2064130
Streptavidin-conjugated Alexa Fluor 546	Invitrogen	Cat# S11225; RRID:AB_2532130
Alexa Fluor 647 anti-rat	Invitrogen	Cat# A21247; RRID:AB_141778
Rabbit anti-PHA-L	Vector Laboratories	Cat# AS-2300; RRID:AB_2313686
Mouse anti-GFP	Invitrogen	Cat# A11120; RRID:AB_221568
Rabbit anti-GFP	Invitrogen	Cat# A11122; RRID: AB_221569
Guinea pig anti-NeuN	Millipore	Cat# ABN90P; RRID:AB_2341095
Mouse anti-NeuN	Millipore	Cat# MAB377; RRID:AB_2298772
Cy3-Streptavidin	Jackson ImmunoResearch	Cat# 016-160-084; RRID:AB_2337244
Alexa Fluor 647 goat anti-rabbit IgG	Jackson ImmunoResearch	Cat# 111-605-144; RRID:AB_2338078
Cy2 goat anti-mouse IgG	Jackson ImmunoResearch	Cat# 111-225-144; RRID: AB_2338021
DyLight 405 goat anti-guinea pig IgG	Jackson ImmunoResearch	Cat# 106-475-003; RRID: AB_2337432

**Bacterial and virus strains**		

AAV5-CaMKIIa-hChR2(H134R)-EYFP	UNC vector core	Karl Deisseroth virus stock
AAV5-CaMKIIa-hChR2(H134R)-EYFP	Addgene	Cat# 26969-AAV5
AAV2-Ef1a-DIO-hChR2(H134R)-EYFP	UNC vector core	Karl Deisseroth virus stock
AAV1-Syn1(S)-FLEX-tdTomato-T2A-SypEGFP	Addgene	Cat# 51509-AAV1
AAV9.CaMKII 0.4.Cre	Addgene	Cat# 105558-AAV9

**Chemicals, peptides, and recombinant proteins**		

Biotinylated dextran amine	Invitrogen	Cat# D1956; RRID:AB_2307337
Phaseolus vulgaris leucoagglutinin	Vector Laboratories	Cat# L-1110; RRID:AB_2336656
Biocytin	Sigma-Aldrich	Cat# B4261
DAPI	Carl Roth	Cat# 6335.1

**Deposited data**		

Data for the propagation of SPW-R to LVI and histological data for the tracing of hippocampal projections	heiDATA	https://doi.org/10.11588/data/EDAJD7

**Experimental models: Organisms/strains**		

Mouse C57BL/6N	Charles River Japan SLC	Strain code: 027
Mouse PV-Cre.tdTomato	Jackson Laboratory	B6.129P2-Pvalb^tm1(cre)Arbr^/J x B6.Cg-Gt(ROSA)26Sor^tm14(CAG-tdTomato)/Hze^/J
Mouse Rbp4-Cre	MMRRC	B6.FVB/CD1-Tg(Rbp4-cre)KL100Gsat/Mmucd

**Software and algorithms**		

CorelDRAW	Corel Corporation	X4
ImageJ/Fiji	NIH	https://imagej.net/Fiji; RRID:SCR_002285
GraphPad	InStat	V3.10 http://graphpad.com/; RRID:SCR_000306
MATLAB	The Mathworks, Inc	https://www.mathworks.com/; RRID: SCR_001622
SigmaPlot	Systat	V11.0 http://www.sigmaplot.com/products/sigmaplot/; RRID:SCR_003210
Origin	OriginLab	Origin 7G; http://microcal-origin.software.informer.com/; RRID:SCR_002815

### Experimental model and study participant details

#### Animals

Characterization of SPW-R propagation to MEC LVI and paired recordings between excitatory neurons were performed in brain slices from 4 to 8 week old male C57BL/6N mice. Hippocampal projections to MEC were anatomically investigated in adult male C57BL/6N mice and electrophysiologically verified in 9–12 week old male C57BL/6N or B6.129P2-Pvalb^tm1(cre)Arbr^/J x B6.Cg-Gt(ROSA)26Sor^tm14(CAG-tdTomato)/Hze^/J (PV-Cre.tdTomato) mice. Paired recordings between excitatory and FS interneurons were carried out in 4–8 week old male PV-Cre.tdTomato mice and connectivity between LVa and LVI was tested in 9–12 week old male B6.FVB/CD1-Tg(Rbp4-cre)KL100Gsat/Mmucd (Rbp4-Cre) mice. C57BL/6N mice were purchased from JapanSLC (Shizuoka, Japan) or Charles River Laboratories (Sulzfeld, Germany). Animals were housed on a 12 h light/dark cycle with *ad libitum* access to food and water. All animal experiments were approved either by the state government of Baden-Württemberg (Projects G206/20 and G58/21) or Tohoku University Center for Laboratory Animal Research (Projects 2017LsA-017 and 2017LsA-018) and were conducted in compliance with German law, Tohoku University Guidelines for Animal Care and Use and the European Communities Council Directive (2010/63/EU).

### Method details

#### Preparation of mouse brain slices

Mice were sacrificed under deep CO_2_-induced anesthesia. After decapitation, brains were rapidly removed and placed in an ice-cold oxygenated cutting solution containing (in mM): 140 K-gluconate, 15 Na-gluconate, 4 NaCl, 10 HEPES and 0.2 EGTA, saturated with carbogen gas (95% O_2_ and 5% CO_2_, pH 7.3). Slices were cut using a vibratome slicer (Leica VT1200S, Nussloch, Germany) at a thickness of 450 μm for SPW-R recordings, 350 μm for optogenetic experiments and 300 μm for paired recordings. To best preserve axonal connectivity, for SPW-R recordings the brain was cut at ∼15° rostrally from the horizontal plane,^15^^,^^29^ whereas for paired recordings the top of the brain was removed at ∼25–30° caudally from the horizontal plane before cutting. For optogenetic experiments, the cutting angle was not adjusted. After cutting, slices used for SPW-R recordings were transferred to a Haas-type interface chamber,^48^ where they were continuously superfused with carbogen-saturated artificial cerebrospinal fluid (ACSF) containing (in mM): 124 NaCl, 3 KCl, 1.6 CaCl_2_, 1.8 MgSO_4_, 10 glucose, 1.25 NaH_2_PO_4_ and 26 NaHCO_3_ (pH 7.4 at 34°C) at a rate of 1.5–2 mL/min. In all other cases, slices were incubated for 20 min at 34°C in carbogen-saturated ACSF and subsequently stored in room temperature (RT) ACSF. Before the start of electrophysiological recordings, slices were allowed to recover for a minimum of 1 h or 2 h in the case of SPW-R experiments.

#### Paired recordings

Individual slices were transferred to a submerged recording chamber, which was continuously superfused with oxygenated ACSF at 32 ± 1°C. LVI, LVb and LVa excitatory neurons were identified with an upright microscope (BX-51WI, Olympus, Japan) at 40× magnification using infrared differential interference contrast (IR-DIC) microscopy. FS interneurons in slices from PV-Cre.tdTomato mice were located by their fluorescence when illuminated by a 565 nm LED (M565L3, ThorLabs, Newton, USA) through the 40×/0.8-NA objective. Paired whole-cell patch clamp recordings between excitatory neurons were performed using borosilicate glass pipettes with a resistance of 3–4 MΩ, filled with a K-based intracellular solution containing (in mM): 144 K-gluconate, 4 NaCl, 10 HEPES, 4 Mg-ATP, 0.3 Na-GTP and 10 Na_2_-phosphocreatine (pH 7.3, KOH, calculated liquid junction potential −17 mV). Paired recordings between excitatory and FS interneurons were made with a K-based intracellular solution containing an elevated concentration of Cl^−^ (in mM): 110 K-gluconate, 30 KCl, 8 NaCl, 10 HEPES, 4 Mg-ATP, 0.3 Na-GTP and 10 Na_2_-phosphocreatine (pH 7.3, KOH). All recordings were performed in current-clamp mode at resting membrane potential (RMP) with the bridge balanced. Presynaptic cells were stimulated with a 10 Hz train of 5 suprathreshold current pulses, repeated every 5 s. A total of 50 consecutive sweeps were collected and averaged to analyze postsynaptic responses. Data were acquired using an ELC-03XS and an SEC-05X amplifier (npi electronics, Tamm, Germany) connected to a POWER1401 mkII analog-to-digital converter (Cambridge Electronic Design (CED), Cambridge, UK). Voltages were low-pass filtered at 3 kHz, digitized at 20 kHz and saved for storage and offline analysis using Signal4 software (CED).

During recordings, LVI excitatory neurons were preliminarily identified based on their location, shape of cell body and firing properties (see below). Intrinsic firing behavior together with input resistance was analyzed by injecting incremental current pulses (25 pA steps, 500 ms) through the recording electrode and measuring the resulting voltage deflections. RMP was measured in current-clamp mode without injecting current and without correcting for liquid junction potential. All intrinsic properties were measured using the low Cl-intracellular solution. LVb and LVa excitatory neurons were identified based on their location, shape of cell body and firing properties as previously described.^15^ FS interneurons were identified based on their characteristic non-adapting high-frequency firing (Figure S3B). During recordings, cells were filled with biocytin (1–5%, Cat. no. B4261, Sigma-Aldrich, Taufkirchen, Germany) which was later immunolabeled to determine cell morphology and location. LVI excitatory neurons typically had a large cell body, horizontally oriented dendrites without a clear apical dendrite and a distinct delayed firing pattern without a sag (Figures 1B–1D and Table S1). This made them clearly distinguishable from neighboring LVb cells, which typically feature a smaller cell body, a prominent apical dendrite and fire without an extended delay.^12^ The axonal arbors of all recorded cells were carefully examined and cells with truncated axons excluded from connectivity analysis as presynaptic neurons. The location of neurons within layers VI and V was further verified by immunolabeling for the transcription factor Ctip2, which marks neurons in a region corresponding to sublayer Vb.

#### Simultaneous SPW-R and whole-cell recordings

Slices from the interface chamber were transferred to a modified submerged double perfusion chamber,^49^ perfused with ACSF at a rate of 9–10 mL/min at 32 ± 1°C. Under these conditions, we observed spontaneously occurring SPW-R that could be reliably observed for at least 2 h.^22^ Extracellular local field potentials were recorded from stratum pyramidale of hippocampal area CA1 using ACSF-filled borosilicate glass pipettes (Cat. no. GB200F-10, Science Products, Hofheim, Germany) with a tip diameter of 3–5 μm. Field potentials were amplified 100×, low-pass filtered at 2 kHz and high-pass filtered at 0.3 Hz with an EXT 10-2F amplifier (npi electronics). Filtered signals were digitized at 20 kHz with the POWER1401 mkII analog-to-digital converter and stored for offline analysis using Spike2 (v7) software (CED). SPW-R in CA1 occurred at 1.8 Hz [1.3, 2.1; *n* = 16] and had a median amplitude of 0.2 mV [0.13, 0.28; *n* = 16]. The vast majority of sharp waves were superimposed by ripples with a leading frequency of 227 Hz [222, 240; *n* = 16]. These values are similar to our previous data.^15^ LVI excitatory neurons were identified with the BX-51WI microscope at 40× magnification using IR-DIC microscopy. Whole-cell patch clamp recordings were performed using borosilicate glass pipettes with a resistance of 3–4 MΩ filled with the same K-based low Cl^−^ intracellular solution as before. Excitatory postsynaptic currents (EPSCs) were recorded in voltage-clamp mode at a holding potential of −74 mV. Baseline activity was monitored for at least 10 min. Whole-cell series resistance was not compensated and closely monitored during recordings. Recordings that showed a series resistance change of >20% were discarded. Data were acquired using the ELC-03XS amplifier connected to the POWER1401 mkII analog-to-digital converter. Currents were low-pass filtered at 8 kHz, digitized at 20 kHz and saved for offline analysis using Spike2 (v7) software.

#### Surgical procedures and tracer/virus injections

General surgical procedures and the *in vivo* delivery of anterograde tracers were described previously.^14^ Briefly, for anterograde tracing experiments either 2.5% phaseolus vulgaris leucoagglutinin (PHA-L; Vector Laboratories, #L-1110) or 3.5–5.0% 10 kDa biotinylated dextran amine (BDA; Invitrogen, #D1956) was injected iontophoretically for 15 min. Alternatively, 200 nL of an adeno-associated virus (AAV) cocktail, consisting of AAV1-Syn1(S)-FLEX-tdTomato-T2A-SypEGFP (1.8 × 1013 GC/mL, 133 nL, Addgene #51509) and AAV9.CaMKII 0.4.Cre (2.1 × 1013 GC/mL, 67 nL, Addgene #105558), was pressure injected using a glass micropipette connected to a Hamilton microsyringe. For electrophysiological experiments involving injections into the hippocampus, 70–100 nL of AAV5-CaMKIIa-hChR2(H134R)-EYFP (UNC vector core, Karl Deisseroth virus stock/Addgene #26969) was injected into either the ventral (AP = −2.9 mm; ML = ±3.4 mm; DV = −4.0 mm) or dorsal hippocampus (AP = −1.5 mm; ML = ±1.2 mm; DV = −1.25 mm) at a rate of 100 nL per minute using a stainless steel needle (NF33BV, inner tip diameter = 115 μm, WPI, Sarasota, USA) connected to a 10 μL NanoFil Syringe (WPI). For experiments in Rbp4-Cre mice, 150 nL of AAV2-Ef1a-DIO-hChR2(H134R)-EYFP (UNC vector core, Karl Deisseroth virus stock) was injected under the same conditions into either ventral (AP = −4.3 mm; ML = ±3.25 mm; DV = −4.0 mm) or dorsal (AP = −4.4 mm; ML = ±3.25 mm; DV = −2.6 mm) MEC LVa. Following each injection, the pipette was left in place for another 10 min before being withdrawn. The wound was sutured and the animal was monitored for recovery from anesthesia, after which it was returned to its home cage.

#### Optogenetically induced postsynaptic responses

Individual slices were transferred to the recording chamber and LVI excitatory or FS interneurons identified as described above. Recordings were performed using borosilicate glass pipettes with a resistance of 3–4 MΩ filled with the K-based low Cl-intracellular solution. For NMDAR current recordings, a Cs-based intracellular solution was used containing (in mM): 144 Cs-gluconate, 4 CsCl, 10 HEPES, 4 Mg-ATP, 0.3 Na-GTP and 10 Na_2_-phosphocreatine (pH 7.3, CsOH, calculated liquid junction potential −17 mV). Axonal fibers of hippocampal or LVa pyramidal cells expressing hChR2 were excited through the 40x/0.8-NA objective using a TTL-controlled blue LED (470 nm, M470L4, ThorLabs). Light intensity was increased at regular intervals (as indicated in Figures 3E, 3F, 4E and 4F) and at each intensity a 10 Hz train of five 1 ms pulses was repeated three times. Alternatively, the 10 Hz train of five 1 ms pulses was repeated ten times at maximum light intensity (11.7 mW/mm^2^). Light-evoked EPSCs were recorded in voltage-clamp mode at a holding potential of −70 mV. To analyze postsynaptic responses, individual sweeps at different light intensities were averaged. Whole-cell series resistance was not compensated and closely monitored during recordings. Recordings showing a change >20% were discarded. Data were acquired as described before and saved for offline analysis using Signal4 and Spike2 (v7) software. Currents were low-pass filtered at 8 kHz and digitized at 20 kHz.

#### Immunohistochemistry of recorded slices

Slices containing biocytin-filled cells were fixed in 4% paraformaldehyde (PFA) in phosphate buffer (PB) for 30–45 min at RT and stored in phosphate buffered saline (PBS) (pH 7.4) at 4°C. For staining, slices were pretreated in blocking solution (5% goat serum and 0.3% Triton X-100 in PBS) for 2 h at RT, followed by washing in PBS (3 × 15 min) and an overnight incubation (>16 h at RT) with the primary antibody (1:1000, rat anti-Ctip2, Abcam #ab18465) diluted in antibody solution (1% goat serum and 0.2% Triton X-100 in PBS). The next day, slices were washed in PBS (3 × 15 min) and treated with the secondary antibodies (streptavidin-conjugated Alexa Fluor 546 (1:1000, Invitrogen #S11225) and Goat anti-Rat IgG conjugated to Alexa Fluor 647 (1:1000, anti-rat, Invitrogen #A21247)) in the antibody solution for 2 h at RT. Slices were then washed in PBS (3 × 15 min) and incubated with 4,6-diamidino-2-phenylindole (DAPI; 1:10 000, Carl Roth, Germany) for 2 min at RT. Finally, slices were rinsed in PBS and embedded in Mowiol 4–88 (Sigma-Aldrich, Taufkirchen, Germany). Confocal image stacks were collected with a Nikon AX confocal microscope (Nikon Imaging Center at Heidelberg University) at 2048x2048 pixel resolution (2 μm z-steps) using 4x (0.13 NA), 10x (0.45 NA) or 20x (0.75 NA) objectives in air. Multiple confocal images were merged as maximum intensity projections and analyzed with ImageJ/Fiji (Wayne Rasband, NIH, USA, open source).

#### Immunohistochemistry of tracing samples

Ten days after tracer or 3–4 weeks after viral injections, the injected animals were anesthetized with isoflurane and euthanized with a lethal intraperitoneal injection of pentobarbital (100 mg/kg). The animals were subsequently transcardially perfused, first with Ringer’s solution (0.85% NaCl, 0.025% KCl, 0.02% NaHCO_3_) and then with 4% PFA in 0.1 M PB. Brains were removed from the skull, post-fixed in PFA overnight, and put in a cryo-protective solution containing 20% glycerol and 2% dimethylsulfoxid (DMSO) diluted in 0.125 M PB. A freezing microtome was used to cut the brains into 40-μm-thick sections in the sagittal plane, which were collected in six equally spaced series for processing. To visualize PHA-L, sections were stained with a primary (1:1000, rabbit anti-PHA-L, Vector Laboratories AS-2300) and a secondary antibody (1:400, Alexa Fluor 647 goat anti-rabbit IgG, Jackson ImmunoResearch #111-605-144), while BDA was visualized with Cy3-streptavidin (1:400, Jackson ImmunoResearch #016-160-084). GFP signals were enhanced with primary (1:500, mouse anti-GFP, Invitrogen #A11120; 1:1000, rabbit anti-GFP, Invitrogen #A11122) and secondary antibodies (1:400, Cy2 goat anti-mouse IgG, Jackson ImmunoResearch #111-225-144; 1:400, Alexa Fluor 647 goat anti-rabbit IgG, Jackson ImmunoResearch #111-605-144). For delineation purposes, sections were stained with primary (1:1000, guinea pig anti-NeuN, Millipore #ABN90P; 1:1000, mouse anti-NeuN, Millipore #MAB377) and secondary antibodies (1:400, DyLight 405 goat anti-guinea pig IgG, Jackson ImmunoResearch #106-475-003; 1:400, Cy2 goat anti-mouse IgG, Jackson ImmunoResearch #111-225-144). For immunofluorescence staining, floating sections were rinsed in PBS containing 0.1% Triton X-100 (PBS-Tx), followed by a 60 min incubation in blocking solution containing 5% goat serum in PBS-Tx at RT. Sections were subsequently incubated with primary antibodies diluted in the blocking solution for 20–40 h at 4°C, washed in PBS-Tx (3 × 10 min), and incubated with secondary antibodies diluted in PBS-Tx for 4–6 h at RT. Finally, sections were washed in PBS (3 × 10 min), mounted on gelatin-coated slides, and coverslipped with Entellan new (Merck Chemicals). Sections were imaged using an automated scanner (Zeiss Axio Scan Z1) as previously described.^14^

### Quantification and statistical analysis

#### Analysis of neuroanatomical tracing samples

To examine hippocampal projections to MEC LVI, we re-analyzed anatomical data from Ohara et al.^14^ using the same methodological approach. Briefly, the distribution of anterogradely labeled hippocampal axons in MEC layers V and VI was quantified in sagittal sections spaced 240 μm apart. MEC was divided into columnar bins by first dividing layer IV into 100 μm wide bins and then extending the bins to layers V and VI. Fluorescence intensity of immunohistochemically labeled axons within each bin was quantified using ImageJ/Fiji (Wayne Rasband, NIH, USA, open source) and in each sample the resulting intensity values for all bins were normalized to the bin with maximum intensity. To compare labeling differences between MEC layers VI, Vb, and Va, we subsequently summed the normalized fluorescence intensities of bins within each layer and then calculated the proportion of labeled fibers in MEC layers VI, Vb, and Va among all labeled fibers.

#### Analysis of electrophysiological data

Paired recording data were analyzed from average traces using Signal4. PSP amplitudes were defined as the difference between PSP peak and event-free baseline before PSP onset and were measured for all PSPs of the five-stimulation-train by manually placing horizontal cursors. Latency values were measured from the first PSP of the five-stimulation-train and represent the time interval between the peak of the presynaptic AP and the onset of the postsynaptic PSP. Cells with an RMP more positive than −55 mV or a series resistance >30 MΩ were excluded from analysis.

For the analysis of optogenetic recordings, the first light-evoked EPSC of the five-pulse-train, averaged across the individual sweeps, was used. EPSC amplitudes were defined analogously to PSPs and were similarly measured by manually placing horizontal cursors. Latencies were measured from the onset of light pulse to the onset of EPSC. Responses with a latency <3.5 ms were considered monosynaptic and responses >3.5 ms polysynaptic.^14^^,^^50^ In fact, across our optogenetic experiments nearly all recorded cells could be divided into one of two categories with a latency either <3.0 ms or >4.0 ms (Figures S2B and S2C), further supporting a clear distinction between mono- and polysynaptic responses based on latency. Latencies and 20%–80% rise times were measured at maximum light intensity. When held at −70 mV, cells with a holding current > 300 pA or a series resistance >30 MΩ were discarded.

Field potential data were analyzed using custom routines in MATLAB (The Mathworks, Natick, USA). Sharp waves were detected from low-pass filtered raw data (60 Hz) by finding local maxima above 0.01 mV within 30 ms time windows. The threshold exceeded baseline noise by four standard deviations, yielding reliable detection of SPW-R, which was confirmed by visual inspection of traces. The number of ripple cycles, represented by the number of ripple troughs that occur during an SPW-R event, was detected by band-pass filtering the signal at 150–300 Hz. The limits for this computation were set to three times the standard deviation of event-free baseline. Simultaneously recorded EPSCs were automatically detected using deconvolution of the raw signal with a Wiener filter (deconvwnr, MATLAB). A single kernel was calculated from a double exponential peak function fitted to PSC kinetics (adapted from Roth et al.^29^ and Pfeiffer et al.^51^). Postsynaptic responses to SPW-R usually contained multiple EPSC events, such that the peak amplitude of the complex waveform was not well suited for quantification. We therefore instead used charge transfer elicited by single SPW-R, calculated as EPSC integral during the time of the event (“area under the curve” in Coulomb (A×s)). Event cross-correlations were calculated between relative time points of detected ripple troughs and PSC onsets. Multiple peaks in the correlation histogram form due to multiple counts of delays associated with preceding and following ripples and may suggest consistent event frequencies between the two signals. Analyses of correlated events were compared to data with shuffled EPSC timepoints. Waveform coherence between SPW-R complexes and cellular synaptic current responses for the quantification of oscillation synchrony was calculated as magnitude-squared coherence in MATLAB (mscohere; Amjad et al.^52^). Before analysis, SPW-R and the respective postsynaptic responses were isolated and concatenated to eliminate contaminations by event-free baseline segments. A coherence threshold of twice the standard deviation of the overall (16 recordings) mean coherence value for frequencies between 350 and 500 Hz was used to separate increased ripple-dependent coherence from unspecific background levels.

#### Statistical analysis

Quantitative electrophysiological data from multiple slices are given as median, data in figures are presented as median, 25th and 75th percentile [P25; P75] and individual values. Anatomical data are presented as mean ± SEM. Statistical analyses were performed using MATLAB, GraphPad (InStat, San Diego, USA) or SigmaPlot (Systat, USA). Two-tailed one-sample t-test was used to compare a single group to a specified value. Two-tailed unpaired t-test or Mann-Whitney U test were used for statistical comparisons of two groups with normal or non-normal distributions, respectively. One-way ANOVA followed by Bonferroni’s post hoc test was used to compare multiple groups with normal distributions. Short-term plasticity in paired recordings was tested with Friedman repeated measures ANOVA on ranks. Pairwise comparisons between real and shuffled SPW-R-evoked EPSC time points were evaluated with the Wilcoxon signed-rank test. Differences in connectivity between the dorsal and ventral MEC in paired recordings were compared using Fisher’s exact test. Regression analysis was performed using simple linear regression or exponential curve fitting in MATLAB (curve fitting toolbox), quantified by correlation coefficient r^2^. A *p* value < 0.05 was regarded as significant and thresholds for significance were set as follows: ∗*p* < 0.05, ∗∗*p* < 0.01 and ∗∗∗*p* < 0.001, ns, not significant.
