# Supplementary material for: Functional and structural organization of medial entorhinal cortex layer VI
Source: iScience. 2025 Mar 12;28(4):112207. doi: 10.1016/j.isci.2025.112207 (PMC11999471; doi:10.1016/j.isci.2025.112207)
Supplement: Document S1. Figures S1–S6 and Tables S1–S3 [file mmc1.pdf]

iScience, Volume *28*

Supplemental information

Functional and structural organization of medial entorhinal cortex layer VI

Märt Rannap, Shinya Ohara, Janis Winterstein, Fabian C. Roth, Andreas Draguhn, and Alexei V. Egorov

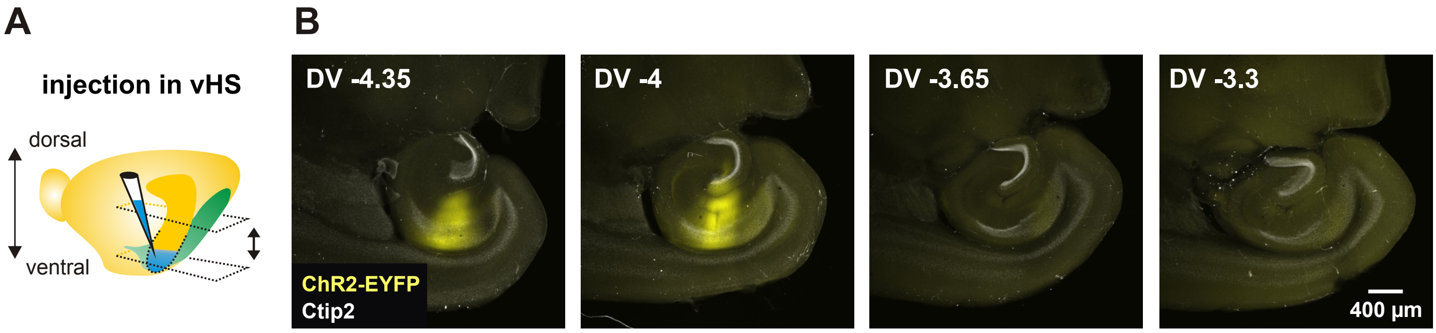

Figure S1. Representative AAV-CaMKIIa-hChR2-EYFP injection site in the ventral hippocampus, related to Figure 3. (A) Illustration of the injection site (blue) in the ventral hippocampus (vHS). The approximate range of horizontal sections shown in B is indicated by dashed lines and arrows. (B) Low magnification confocal images of horizontal sections taken at different dorsoventral levels showing the injection site in ventral CA1. All images have equal calibration, scale bar indicated on the right.

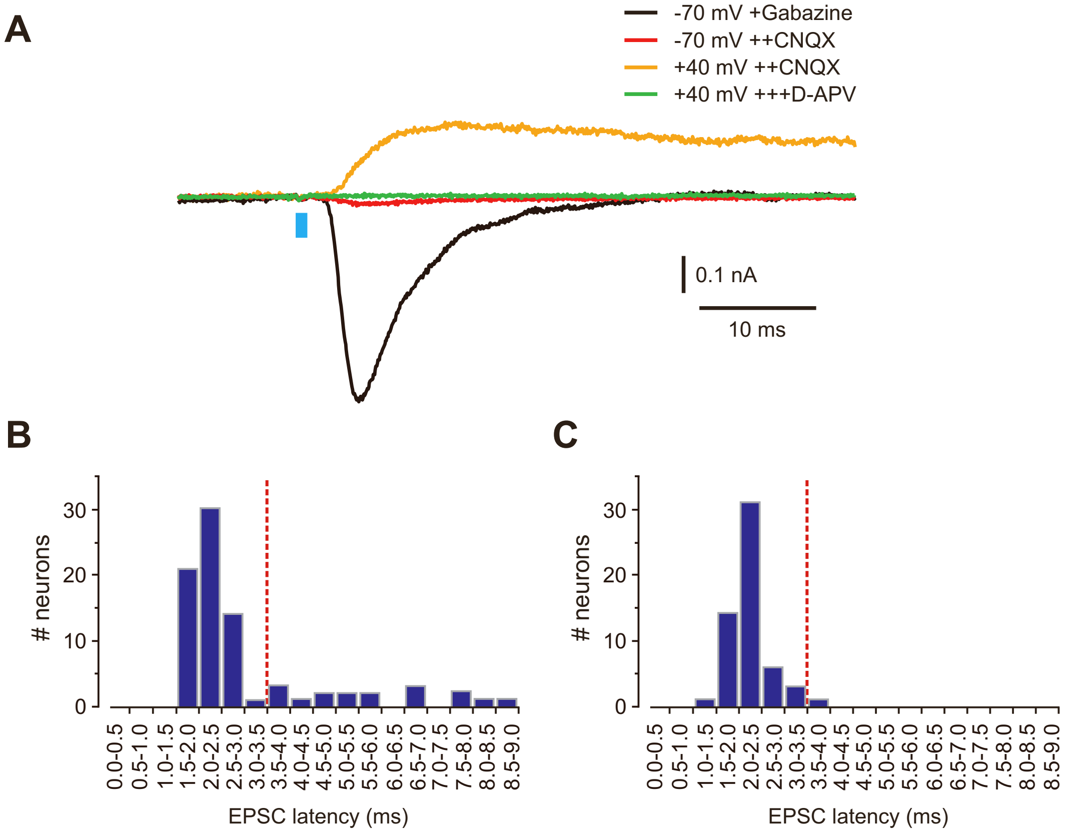

Figure S2. NMDAR currents recorded in the presence of CNQX and gabazine, related to Figure 3, and latency distributions for light-evoked EPSCs across different optogenetic experiments, related to Figures 3, 4, 7, S3 and S5. (A) Representative EPSC traces recorded from an excitatory LVI neuron in response to 1 ms light pulses (vertical blue line). Shown is a baseline EPSC recorded at -70 mV in the presence of 100 µM gabazine (black trace), which was almost entirely blocked by the perfusion of 10 µM CNQX (red trace). With gabazine and CNQX still in the bath, currents could be reliably recorded at +40 mV (orange trace), which were subsequently blocked by the perfusion of 30 µM D-APV (green trace). (B) Histogram showing the combined distribution of light-evoked EPSC latencies for all recorded LVI, LVa and LVb principal neurons from Figures 3 and 4 and LVI FS interneurons from Figures S3 and S5. EPSCs in these experiments were induced by the activation of dorsal or ventral hippocampal axons expressing hChR2-EYFP. The red dashed line indicates the cutoff between putative mono- and polysynaptic responses. (C) Histogram showing the combined distribution of light-evoked EPSC latencies for all recorded LVI and LVb principal neurons from Figure 7. EPSCs in these experiments were induced by the activation of LVa principal cell axons expressing hChR2-EYFP.

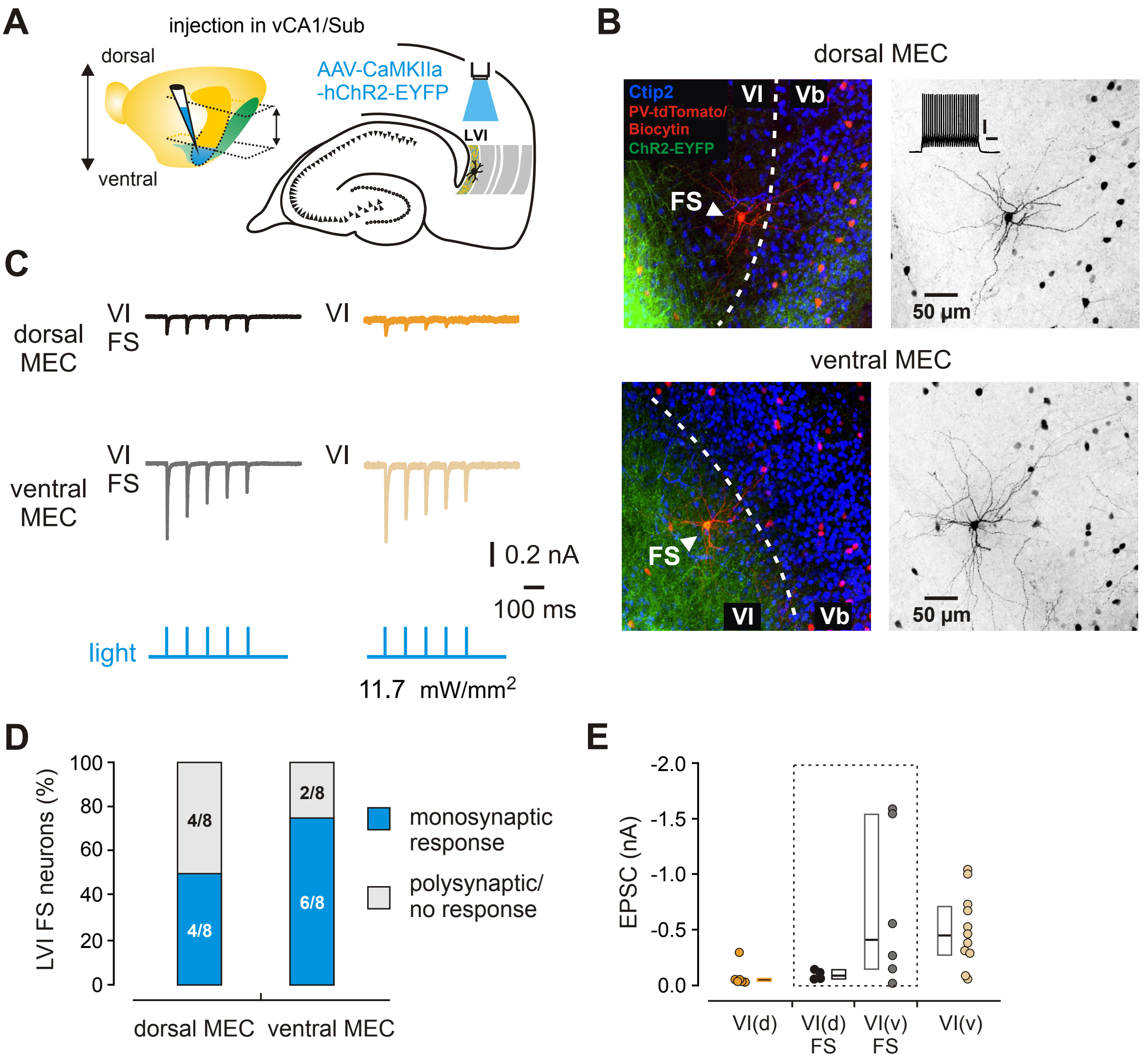

Figure S3. Functional connectivity between the ventral hippocampus and MEC layer VI FS interneurons, related to Figure 3. (A) Left: illustration of the injection site (blue) in the ventral hippocampus of PV-Cre.tdTomato mice with the approximate range of horizontal sections used in experiments indicated by dashed lines and arrows. Right: schematic drawing of a horizontal hippocampal-EC slice showing the position of light stimulation used to activate the axons of ventral hippocampal neurons infected with AAV-CaMKIIa-hChR2-EYFP. (B) Maximum intensity projection confocal images of typical horizontal slices from virus-injected PV-Cre.tdTomato mice (PV cells in red). The images show recorded LVI FS interneurons from the dorsal (top) and ventral MEC (bottom) labeled with biocytin (red), overlaid with Ctip2 labeling and fluorescent staining of hippocampal axons expressing hChR2-EYFP. Right images show the same neurons in black and white contrast. All images have equal calibration, scale bars indicated on the right. Inset shows typical FS cell firing behavior in response to a 0.3 nA current injection, scale bars correspond to 20 mV and 100 ms. (C) Example EPSC traces recorded from LVI FS interneurons in the dorsal (top) and ventral (middle) MEC in response to 1 ms blue light pulses (bottom). For comparison, respective LVI principal cell traces from Figure 3C are shown on the right. (D) Proportion of putative monosynaptic responses from LVI FS cells recorded in the dorsal or ventral MEC. (E) Quantification of EPSC amplitudes from LVI FS interneurons in the dorsal (VI FS(d)) or ventral MEC (VI FS(v)) in response to maximum intensity light pulses (11.7 mW/mm^2, VI FS(d), 4 cells from 3 mice; VI FS(v), 6 cells from 6 mice). Amplitude values for FS cells are compared with respective LVI excitatory neuron amplitudes from Figure 3E. All data are presented as median (black line), 25th and 75th percentiles (box). Circles represent individual values.

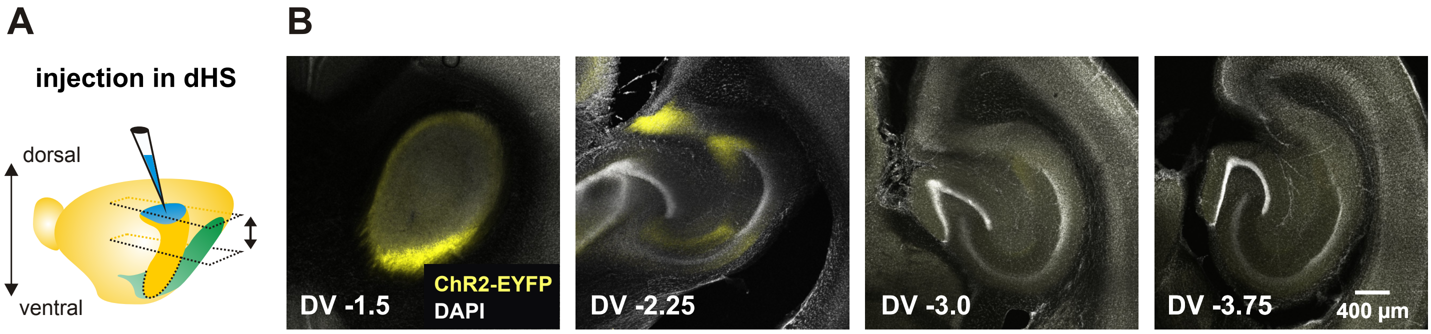

Figure S4. Representative AAV-CaMKIIa-hChR2-EYFP injection site in the dorsal hippocampus, related to Figure 4. (A) Illustration of the injection site (blue) in the dorsal hippocampus (dHS). The approximate range of horizontal sections shown in B is indicated by dashed lines and arrows. (B) Low magnification confocal images of horizontal sections taken at different dorsoventral levels showing the injection site in dorsal CA1. All images have equal calibration, scale bar indicated on the right.

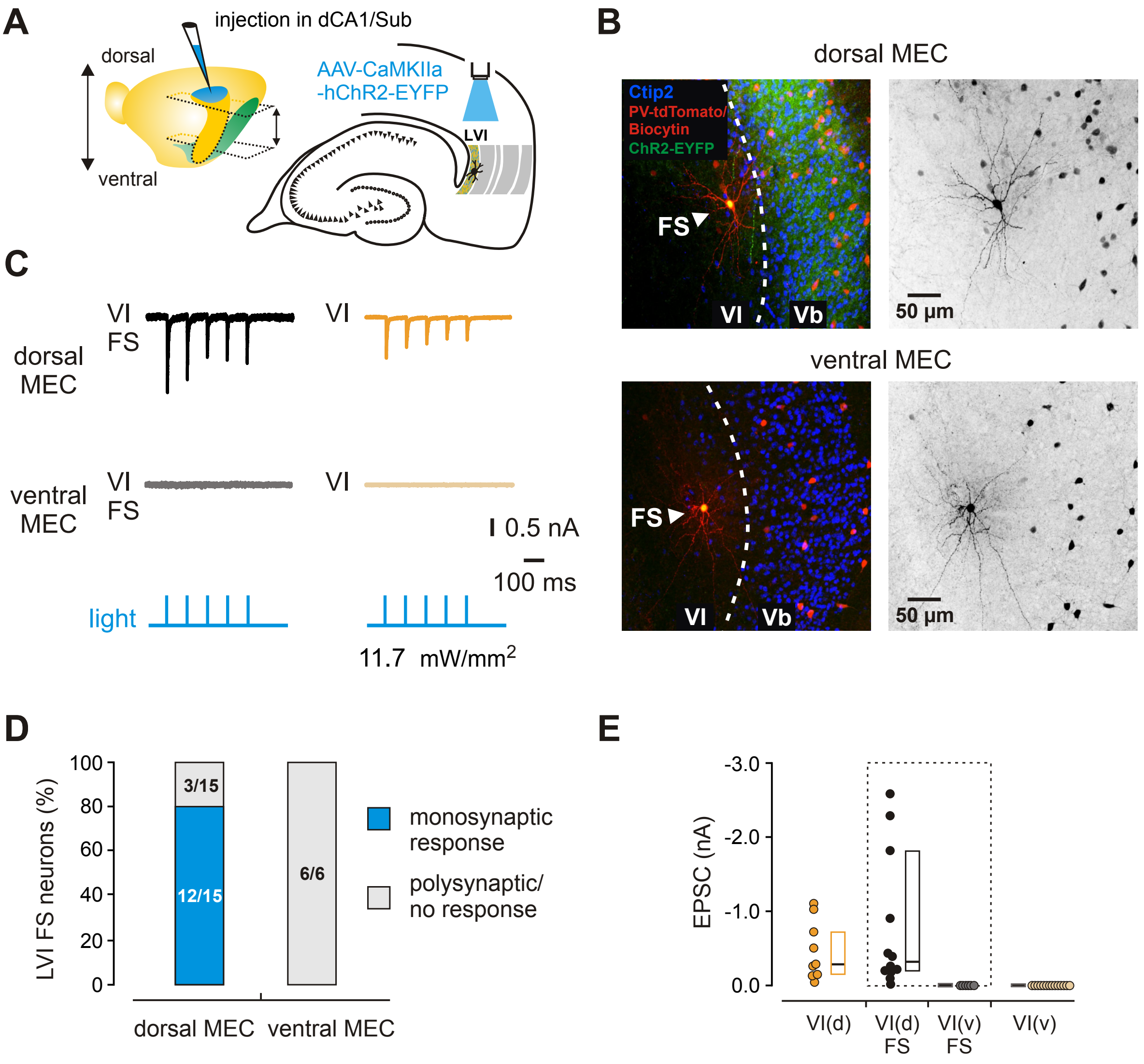

Figure S5. Functional connectivity between the dorsal hippocampus and MEC layer VI FS interneurons, related to Figure 4. (A) Left: illustration of the injection site (blue) in the dorsal hippocampus of PV-Cre.tdTomato mice with the approximate range of horizontal sections used in experiments indicated by dashed lines and arrows. Right: schematic drawing of a horizontal hippocampal-EC slice showing the position of light stimulation used to activate the axons of dorsal hippocampal neurons infected with AAV-CaMKIIa-hChR2-EYFP. (B) Maximum intensity projection confocal images of representative horizontal slices from virus-injected PV-Cre.tdTomato mice (PV cells in red). The images show recorded LVI FS interneurons from the dorsal (top) and ventral MEC (bottom) labeled with biocytin (red), overlaid with Ctip2 labeling and fluorescent staining of hippocampal axons expressing hChR2-EYFP. Right images show the same neurons in black and white contrast. All images have equal calibration, scale bars indicated on the right. (C) Example EPSC traces recorded from LVI FS interneurons in the dorsal (top) and ventral (middle) MEC in response to 1 ms blue light pulses (bottom). For comparison, respective LVI principal cell traces from Figure 4C are shown on the right. (D) Proportion of putative monosynaptic responses from LVI FS cells recorded in the dorsal or ventral MEC. (E) Quantification of EPSC amplitudes from LVI FS interneurons in the dorsal (VI FS(d)) or ventral MEC (VI FS(v)) in response to maximum intensity light pulses (11.7 mW/mm^2, VI FS(d), 12 cells from 5 mice; VI FS(v), 6 cells from 4 mice). Amplitude values for FS cells are compared with respective LVI excitatory neuron amplitudes from Figure 4E. All data are presented as median (black line), 25th and 75th percentiles (box). Circles represent individual values.

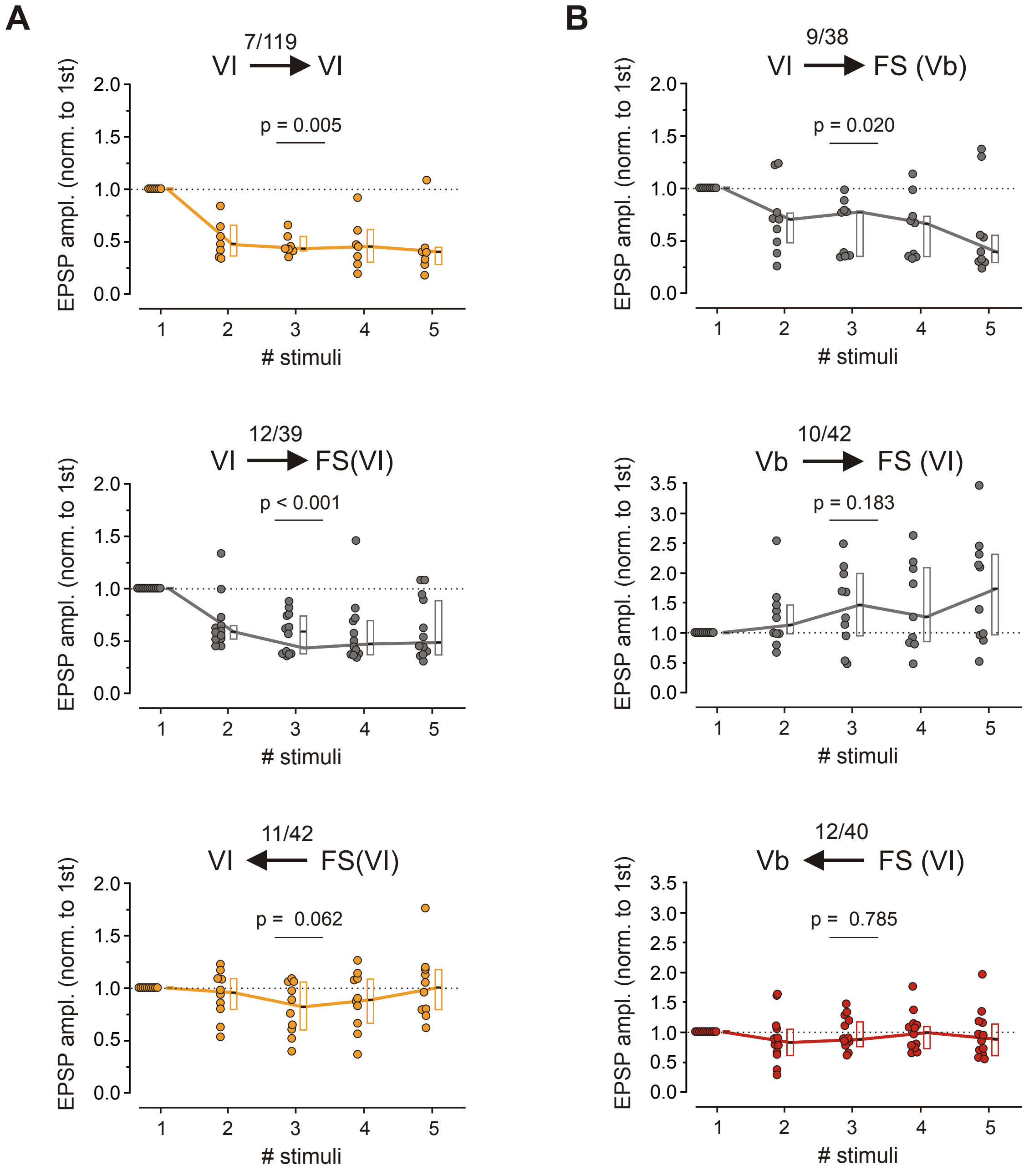

Figure S6. Short-term plasticity of synaptic connections between LVI and LVb excitatory and FS interneurons, related to Figures 5 and 6. (A) Short-term plasticity of synaptic connections between LVI excitatory neurons (top), from LVI excitatory to LVI FS interneurons (middle) and from LVI FS to LVI excitatory cells (bottom). For all connections, PSP amplitudes in response to five consecutive presynaptic APs were normalized to the first PSP amplitude. (B) Same analysis as in A for connections from LVI excitatory to LVb FS cells (top), from LVb excitatory to LVI FS cells (middle) and from LVI FS to LVb excitatory cells (bottom). All data are presented as median (black line), 25th and 75th percentiles (box). Circles represent individual values. Friedman repeated measures ANOVA on ranks.

Table S1. Intrinsic electrophysiological properties of MEC LVI excitatory neurons, related to Figure 1.

	Mean ± SEM (n = 34)
Resting membrane potential (mV)	-72.19 ± 0.84
Input resistance (MΩ)[1]	179.73 ± 11.02
Sag ratio[2]	1.01 ± 0.001
AP threshold (mV)[3]	-38.00 ± 0.75
AP peak (mV)[4]	71.73 ± 0.97
AP half-width (ms)[5]	1.53 ± 0.03
AP 20%-80% rise time (ms)	0.26 ± 0.01
Max rise slope (mV/ms)[6]	175.81 ± 5.44
Max decay slope (mV/ms)[7]	-40.64 ± 1.18
AHP amplitude (mV)[8]	-11.55 ± 0.37
Rheobase current (pA)	116.91 ± 6.31
Latency to 1st AP (ms)[9]	320.34 ± 13.13
Adaptation[10]	0.77 ± 0.03

All AP properties were measured from the first AP at rheobase current. AP, action potential; AHP, afterhyperpolarization.

1. Measured from peak voltage deflection in response to a -25 pA current injection according to Ohm's law
2. Ratio between peak voltage deflection and steady-state voltage following a -200 pA current injection
3. Membrane potential at the point where AP rate of rise reached 20 mV/ms
4. Voltage difference between AP threshold and peak
5. Full width at half maximum
6. Maximum rate of rise during the AP rising phase
7. Maximum rate of decay during the AP decaying phase
8. Peak negative voltage deflection after the AP relative to the threshold
9. Measured from the onset of the current injection to the threshold
10. Time interval between the first two APs divided by the interval between the last two APs, measured from traces with 8 ± 1 APs

Table S2. Differences in local LVI connectivity between the dorsal and ventral MEC, related to Figure 5.

		Connectivity	Amplitude (nA)	Latency (ms)	STP (5th/1st)
VI ↔ VI	dMEC	3 of 55 (5.5%)	0.32 [0.16; 1.13]	2.30 [0.59; 4.46]	0.34 [0.19; 0.44]
		p > 0.999	p = 0.114	p = 0.629	p = 0.629
	vMEC	4 of 64 (6.3%)	0.10 [0.08; 0.17]	1.74 [1.50; 1.85]	0.41 [0.31; 0.91]
VI → FS (VI)	dMEC	7 of 21 (33.3%)	0.18 [0.10; 4.18]	0.55 [0.42; 1.13]	0.89 [0.45; 1.08]
		p = 0.742	p = 0.530	p = 0.530	p = 0.020*
	vMEC	5 of 18 (27.8%)	0.95 [0.25; 2.44]	0.81 [0.59; 1.43]	0.37 [0.33; 0.50]
FS (VI) → VI	dMEC	7 of 24 (29.2%)	0.34 [0.15; 0.48]	0.56 [0.26; 0.64]	1.06 [0.80; 1.18]
		p = 0.731	p = 0.833	p = 0.050	p = 0.667
	vMEC	4 of 18 (22.2%)	0.21 [0.13; 1.04]	0.78 [0.66; 2.43]	0.80 [0.62; 1.76]

Values are presented as median, 25th and 75th percentile [P25; P75]. Amplitude and latency values correspond to the first EPSP of the five stimulation train. Short-term plasticity values represent the amplitude of the last EPSP normalized to the first. dMEC, dorsal MEC; vMEC, ventral MEC; STP, short-term plasticity. Mann-Whitney or Fisher's exact test: *p < 0.05.

Table S3. Differences in LVI-LVb connectivity between the dorsal and ventral MEC, related to Figure 6.

		Connectivity	Amplitude (nA)	Latency (ms)	STP (5th/1st)
VI → Vb	dMEC	1 of 59 (1.7%)	0.04	1.66	0.60
	vMEC	0 of 61			
Vb → VI	dMEC	0 of 58			
	vMEC	0 of 64			
VI → FS (Vb)	dMEC	5 of 19 (26.3%)	0.33 [0.10; 0.36]	0.66 [0.56; 0.94]	0.39 [0.28; 0.93]
		$p > 0.999$	$p = 0.905$	$p = 0.064$	$p > 0.999$
	vMEC	4 of 19 (21.1%)	0.19 [0.16; 0.45]	1.04 [0.75; 2.23]	0.41 [0.29; 1.16]
FS (Vb) → VI	dMEC	0 of 21			
	vMEC	1 of 20 (5.0%)	0.13	1.61	0.74
Vb → FS (VI)	dMEC	5 of 22 (22.7%)	0.22 [0.12; 0.48]	0.46 [0.42; 0.67]	1.39 [0.92; 2.84]
		$p > 0.999$	$p = 0.691$	$p = 0.548$	$p = 0.841$
	vMEC	5 of 20 (25.0%)	0.35 [0.12; 0.61]	0.43 [0.18; 1.59]	2.09 [0.76; 2.23]
FS (VI) → Vb	dMEC	5 of 20 (25.0%)	0.22 [0.05; 0.48]	0.54 [0.48; 0.94]	1.17 [0.93; 1.65]
		$p = 0.731$	$p = 0.639$	$p = 0.048$*	$p = 0.018$*
	vMEC	7 of 20 (35.0%)	0.23 [0.11; 0.40]	1.02 [0.77; 1.47]	0.70 [0.56; 0.94]

Values are presented as median, 25th and 75th percentile [P25; P75]. Amplitude and latency values correspond to the first EPSP of the five stimulation train. Short-term plasticity values represent the amplitude of the last EPSP normalized to the first. dMEC, dorsal MEC; vMEC, ventral MEC; STP, short-term plasticity. Mann-Whitney or Fisher's exact test: *$p < 0.05$.
